# Causal Effects of Circulating Cytokines on the Risk of Psoriasis Vulgaris: A Mendelian Randomization Study

**DOI:** 10.3389/fgene.2022.941961

**Published:** 2022-06-13

**Authors:** Pan Zhao, Jing Zhang, Biyong Liu, Yufei Tang, Lei Wang, Guifeng Wang, Huihui Wu, Chengwei Yang, Xuemei Li, Bo Li

**Affiliations:** Anhui Prevention and Treatment Center for Occupational Disease, Anhui No. 2 Provincial People’s Hospital, Hefei, China

**Keywords:** psoriasis vulgaris, cytokines, causal effect, mendelian randomization, RANTES

## Abstract

**Background:** Psoriasis vulgaris is an inflammatory skin disease. Observational studies have shown associations between circulating cytokine levels and psoriasis vulgaris. But the causal relationship between circulating cytokine and psoriasis vulgaris remains elusive.

**Methods:** To assess the causal effects of cytokine levels on the risk of psoriasis vulgaris and vice versa, we performed a two-sample Mendelian randomization (MR) study by using the inverse-variance weighted (IVW), weighted median, and Mendelian randomization pleiotropy residual sum and outlier (MR-PRESSO) in genome-wide association summary statistics of 41 circulating cytokines in up to 8,293 individuals and psoriasis vulgaris in 399,883 individuals.

**Results:** We identified that increasing RANTES level induced an elevated risk of psoriasis vulgaris in IVW (*β* = 0.33, S.E. = 0.12, *p* = 0.006). This causal effect showed consistency across the weighted median (*β* = 0.35, S.E. = 0.15, *p* = 0.022) and MR-PRESSO method (*β* = 0.33, S.E. = 0.11, *p* = 0.028).

**Conclusions:** Our results suggest a potential causal effect of elevated RANTES concentration on the increased risk of psoriasis vulgaris.

## Introduction

Psoriasis vulgaris is a chronic inflammatory skin disease, affecting about 2% of the population around the world (von Csiky-Sessoms and Lebwohl, 2019; Rendon and Schäkel, 2019). The prevalence of psoriasis vulgaris varies across regions, ranging from 0.05 to 0.47% in Asia to 2–3% in Europe and the United States (Hawkes et al., 2017; Kaufman and Alexis, 2018). It is clinically characterized by erythema lesions covered with pearly scales (Hawkes et al., 2017; Rendon and Schäkel, 2019). The exact cause of psoriasis vulgaris is not well understood. Observational studies have shown associations between psoriasis vulgaris and circulating cytokines such as IL-23, IL-17 and TNF-α (de Alcantara et al., 2021). However, the causal relationship between cytokines and psoriasis vulgaris remain unresolved.

Since observational studies are susceptible to reverse causality and confounding factors, it is unable to provide convincing evidence for causality alone (Smith and Ebrahim, 2004). For instance, elevated IL-22 levels have been observed in both skin lesions and blood, the strategy of blocking IL-22 in the treatment of psoriasis failed in a clinical trial (Chiricozzi et al., 2018). Studies show that plasmacytoid dendritic cells in psoriatic lesions produce IFN-α, suggesting a key role of IFN-α in the pathogenesis of psoriasis (Gilliet et al., 2004; Nestle et al., 2005). However, a randomized trial failed to provide evidence that targeting IFN-α improved clinical symptoms in patients with plaque psoriasis (Bissonnette et al., 2010). Many studies detect an increasing level of IL-17 in the occurrence and development of psoriasis (Harper et al., 2009; Johansen et al., 2009; Ramirez-Carrozzi et al., 2011). But in an experimental study, IL-17 restored the function of keratinocytes and played a protective role in the development of psoriasis (Kanda et al., 2005; Deng et al., 2016).

Mendelian randomization (MR) uses genetic variation that are strongly associated with a possible exposure as instrumental variables (IVs) to assess the causality of the given exposure on an outcome of interest (Emdin, et al., 2017). Compared with observational studies, MR is immune from reverse causality and confounding factors. In past decade, genome-wide association studies (GWAS) have identified tens of thousands of genetic variants robustly associated with complex traits (Hemani, et al., 2018). These findings reveal the genetic underpinning of complex traits. The associated genetic variant can be used as IVs and facilitate the application of MR to identify disease’s causal factors (Davey Smith and Hemani, 2014).

To better understand the causal role of cytokines in the development of psoriasis vulgaris and to search for disease prevention and treatment targets of potential, we performed a two-sample MR to evaluate the causal relationship between circulating cytokines concentrations and the risk of psoriasis vulgaris. We identified single nucleotide polymorphisms (SNPs) from GWAS meta-analysis for 41 circulating cytokines in 8,293 Finns as IVs. We evaluated the causal effect of circulating cytokines on psoriasis vulgaris with SNPs which were identified, and the genome-wide association statistics for psoriasis vulgaris in the United Kingdom biobank. Our study suggests a potential causal association between circulating RANTES levels and the risk of psoriasis vulgaris. This finding helps identify a potential target for the intervention of psoriasis.

## Materials and Methods

### Genome-Wide Association Summary Statistics

We accessed genome-wide association summary-level statistics for 41 circulating cytokines and psoriasis vulgaris. In brief, the summary-level data for 41 cytokines included ∼ 10.7 million SNPs and 8,293 Finnish individuals from the Cardiovascular Risk in Young Finns study, FINRISK 1997, and FINRISK 2002 (Ahola-Olli et al., 2017). The cytokine levels were quantified by Bio-Rad’s premixed Bio-Plex Pro Human Cytokine 27-plex Assay and 21-plex Assay, and Bio-Plex 200 reader with Bio-Plex 6.0 software. Genotype imputation inference was based on reference haplotypes from the 1000 Genomes Project Phase 1 (1000 Genomes Project Consortium et al., 2010). The single-variant association was accomplished through a linear regression between cytokine levels and SNPs after adjusting for age, sex, and body mass index (Ahola-Olli et al., 2017).

We used genome-wide association statistics for psoriasis vulgaris in the United Kingdom biobank (Sudlow et al., 2015). The United Kingdom Biobank defined psoriasis vulgaris using International Classification of Diseases (ICD) L40.0 based on the medical records and questionnaire data for each participate. The United Kingdom biobank data contains 1,684 psoriasis vulgaris cases and 398,199 controls of White British. GWAS was accomplished between the risk of psoriasis vulgaris and 31.3 million SNPs through SAIGE (Sudlow et al., 2015; Zhou et al., 2018). About 8.4 million SNPs were overlapped with the GWAS for circulating cytokines. No ethical approval was required.

### Identification of IVs

The IVs for MR have three assumptions: 1) IVs must be strongly associated with exposure. 2) IVs are not associated with any known confounders related to outcome or exposure. 3) IVs cannot affect the outcome in other ways except through exposure (Lawlor et al., 2008). To identify IVs for MR analysis, we performed linkage disequilibrium (LD) pruning in Plink 2.0 in the 8.4 million overlapping SNPs at *P* < 1 × 10^−6^ in cytokine GWAS summary statistics using LD *r* ^2^ < 0.1 in distance≥500 kilobases (kb) (Machiela and Chanock, 2015; Sobota et al., 2015). After screening, 420 SNPs associated with 40 cytokines were used for further analyses as IVs. To attain a stable assessment, we restricted our MR analyses to 35 of the 41 cytokines with the number of IVs ≥3.

### MR Analysis

To test the potential causal effects of circulating cytokine levels on the risk of psoriasis vulgaris and vice versa, we ran MR analysis by using three two-sample MR methods: IVW, weighted median, and MR-PRESSO (Burgess et al., 2017; Verbanck et al., 2018). These three methods make different assumptions and deal with the potential horizontal pleiotropy differently. The IVW method integrates causal effects assessment from multiple genetic variants, assuming that all IVs are valid and free of interference from heterogeneity and pleiotropy (Pierce and Burgess, 2013). When at least 50% SNPs are valid instruments, weighted median test is recommended (Bowden et al., 2016). The MR-PRESSO test is appropriate when less than 50% of the genetic variants have a pleiotropic effect (Verbanck et al., 2018). We required the causal effects consistent across all the three methods and used a significance threshold *p* < 0.05 to define a significant causal effect (Burgess et al., 2017; Verbanck et al., 2018).

### Test for Heterogeneity and Pleiotropy

To evaluate the validity of the MR core hypothesis, we performed a series of assessments of heterogeneity and pleiotropy. MR-PRESSO method can test the directional pleiotropy and identify and correct potential outliers (Verbanck et al., 2018). We evaluated horizontal pleiotropy by examining the intercept term of MR Egger’s regression (Bowden et al., 2015). If the intercept term is significantly different from zero at *p* < 0.05, horizontal pleiotropy exists (Bowden et al., 2015). We calculated a Cochran’s Q-statistic to quantify the heterogeneity of causal effects across all IVs (Egger et al., 1997).

## Results

### Selection of IVs

For 35 of the 41 cytokines, we identified at least three genetic variants available at *p* < 1 × 10^–6^ and LD (*r* ^2^ < 0.1, distance≥500 kb). The numbers of IVs for each cytokine are shown in Table 1. The associations of IVs with each circulating cytokine level can be found in Supplementary Table S1.

**TABLE 1:** MR analysis of the causal relationship between RANTES, SDF-1α, MIP-1β and IL-17 levels and psoriasis vulgaris.

Cytokines	No. of SNPs	Association			Heterogeneity	MR-PRESSO global Test
		*β*	S.E.	*P*-Value	*P*-Value	*P*-Value
RANTES						
IVW	6	0.325	0.119	**0.006**	0.55	—
Weighted median	6	0.348	0.153	**0.022**	—	—
MR-PRESSO	6	0.325	0.106	**0.028**	—	0.617
SDF-1α						
IVW	3	−0.533	0.221	**0.016**	0.37	—
Weighted median	3	−0.516	0.267	0.053	—	—
MR-PRESSO	3	—	—	—	—	—
MIP-1β						
IVW	145	−0.047	0.030	0.117	0.02	—
Weighted median	145	−0.096	0.044	**0.028**	—	—
MR-PRESSO	145	NA	NA	NA	—	0.023
IL-17						
IVW	4	0.310	0.381	0.416	0.02	—
Weighted median	4	0.512	0.295	0.082	—	—
MR-PRESSO	4	0.623	0.105	**0.027**	—	0.027

Note: Significant results are shown in bold.

### Causal Effects of Cytokine Levels on the Risk of Psoriasis Vulgaris

**FIGURE 1:**
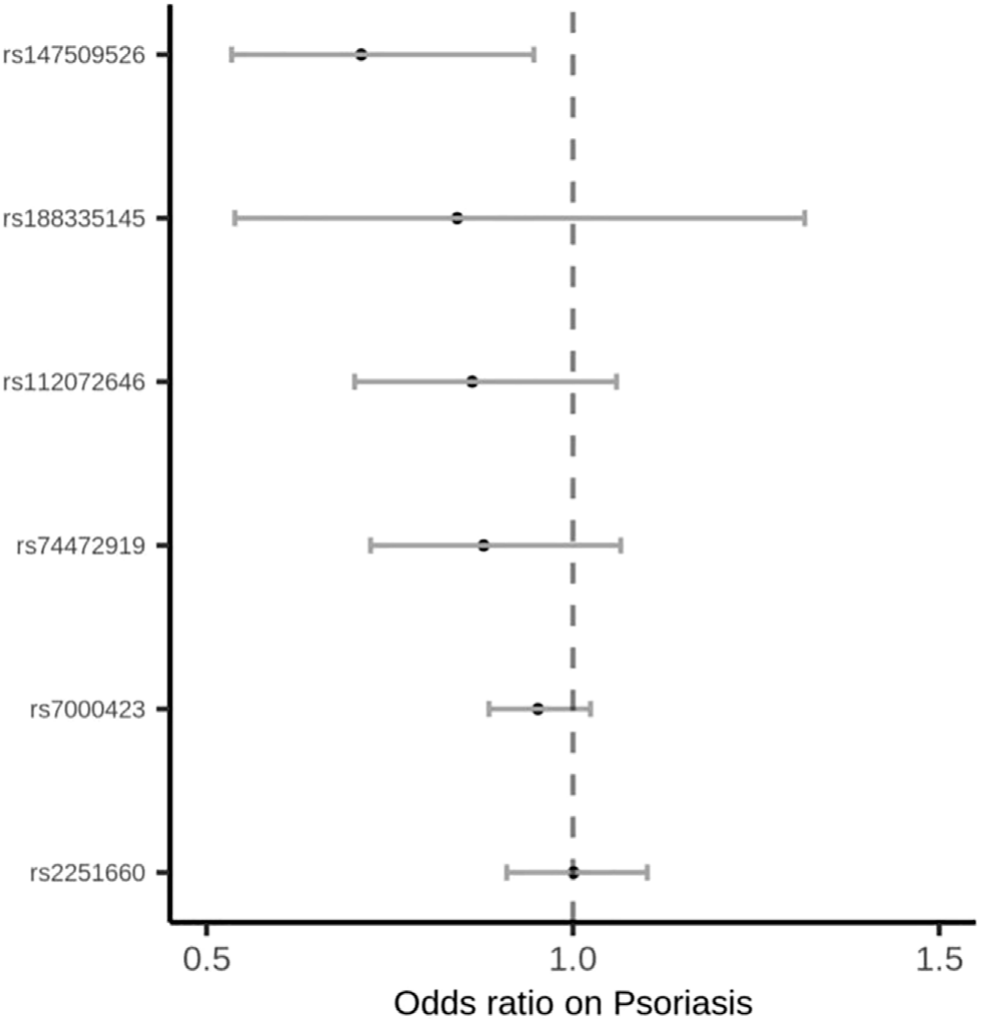
Forest plot of the effects of IVs for RANTES on the risk of psoriasis vulgaris.

**FIGURE 2:**
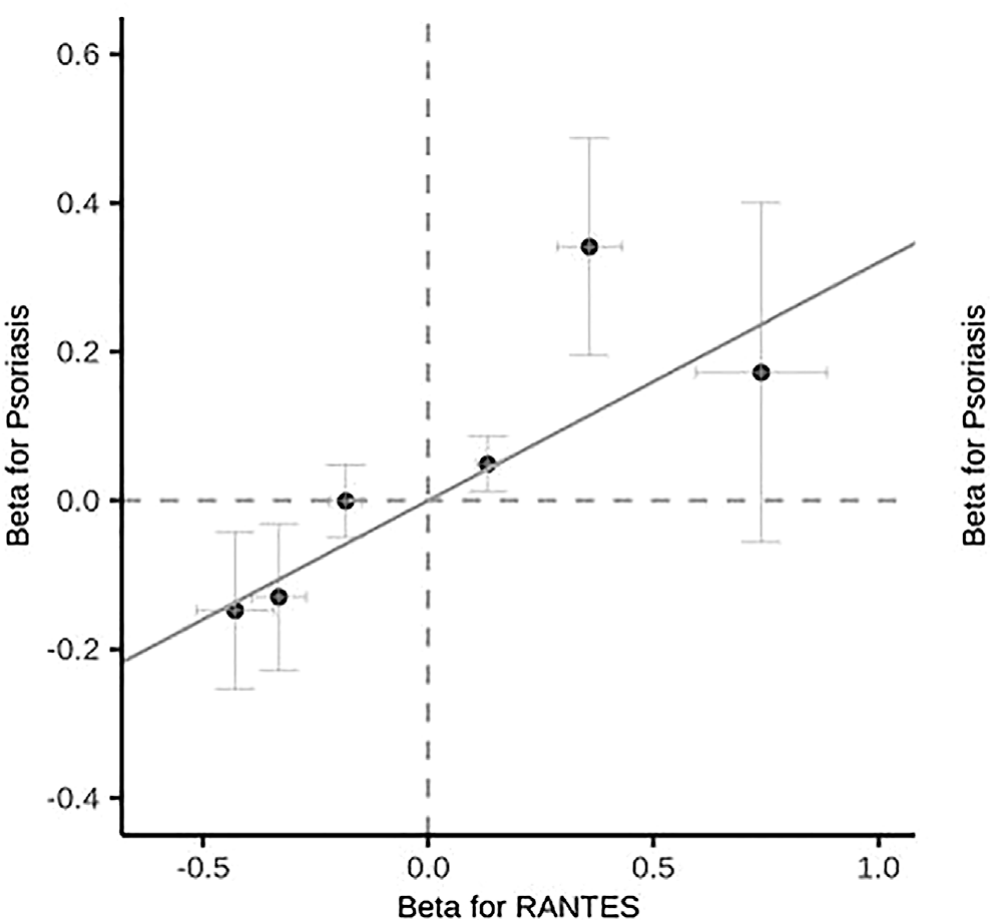
Scatter plot of the effects of IVs on RANTES levels and the risk of psoriasis vulgaris.

Among the 35 cytokines, genetically predicted RANTES, SDF-1α, MIP-1β and IL-17 were associated with the risk of psoriasis vulgaris in at least 1 MR method at the significance threshold (*p* < 0.05; Table 1). MR results of the rest 31 cytokines in our analyses can be found in Supplementary Table S2. In the four cytokines RANTES, SDF-1α, MIP-1β, and IL-17, only the causal effect of RANTES on psoriasis vulgaris was significant in all the 3 MR methods: IVW (*β* = 0.325, S.E. = 0.119, *p* = 0.006), weighted median (*β* = 0.348, S.E. = 0.153, *p* = 0.022), and MR-PRESSO (*β* = 0.325, S.E. = 0.106, *p* = 0.028; Table 1 and Figures 1, 2). We identified six IVs for the MR analysis evaluating the causal effect of RANTES. The causal effects of these six IVs on the risk of psoriasis vulgaris showed no statistically significant heterogeneity (Cochran’s Q-test *p* = 0.55; Table 1). We observed no evidence of horizontal pleiotropy in the MR-PRESSO (Global test *p* = 0.62; Table 1) or the MR-Egger regression (*p* for intercept = 0.69; Supplementary Table S3).

### Causal Effects of Psoriasis Vulgaris on Circulating Cytokine Levels

Reversely, we identified causal effects of the onset of psoriasis vulgaris on 11 circulating cytokine levels including IL-1ra, IL-5, CTACK, MIG, VEGF, IL-8, IL-9, IL-13, IL-12p70, TNF-α, and IL-2 at *p* < 0.05 in at least one of the 3 MR methods based on MR results from Supplementary Table S4. Out of these 11 cytokines, the causal effects of psoriasis vulgaris achieved consistently significant on IL-1ra, IL-5, and MIG in all the 3 MR methods. We did not detect significant heterogeneity of causal effects (Cochran’s Q-test *p* > 0.05; Supplementary Table S4) or horizontal pleiotropy (MR-PRESSO Global test *p* > 0.05 and MR Egger’s regression intercept *p* > 0.05; Supplementary Tables S4, S5).

## Discussion

Studies have shown significant associations between circulating cytokines and psoriasis vulgaris, but the causal relationship between cytokines and psoriasis vulgaris remain unclear. In our study, we leveraged large-scale GWAS for 41 circulating cytokines and psoriasis vulgaris and evaluated the causal relationship between circulating cytokines and psoriasis vulgaris by using three two-sample MR approaches. We identified a causal effect of elevated RANTES levels on the increased risk of psoriasis vulgaris. We also assessed the causal effects of psoriasis vulgaris risk on circulating cytokine concentrations, which found that increased risk of psoriasis vulgaris resulted in elevated circulating levels of IL-1ra, IL-5, and MIG.

Psoriasis vulgaris is characterized by abnormal epidermal hyperplasia and differentiation, with accumulation and infiltration of neutrophils and pro-inflammatory T cells in the *epidermis* and dermis (Chiricozzi et al., 2018). RANTES is a pro-inflammatory chemokine, which not only mediates the infiltration of lymphocytes, granulocytes, natural killer cells, and dendritic cells into the inflammatory lesions, but also activates leukocytes to play a role in the inflammatory response (Appay and Rowland-Jones, 2001; Levy, 2009; Marques et al., 2013). RANTES has been reported to play an important role in leukocyte recruitment to psoriatic skin lesions (Nedoszytko et al., 2014; Zablotna et al., 2016). Studies have shown that RANTES production increased in psoriatic lesions compared with non-lesion skin, and was mainly present in keratinocytes or the intercellular space between keratinocytes in psoriatic lesions, from the middle to the marginal lesions of psoriatic plaques (Rateb et al., 2012; Johansen et al., 2017; Joshi et al., 2019). The expression of RANTES’s receptor CCR5 was enhanced in epidermal T cells, and the number of CCR5 positive cells raised significantly too (de Groot et al., 2007; Joshi et al., 2019). Phototherapy has been reported to inhibit the production of RANTES in patients with psoriasis. After treatment with NB-UVB, the expression of RANTES was significantly reduced (Rateb et al., 2012; Joshi et al., 2019). According to several case-control trials, serum concentrations of RANTES were significantly higher in patients with psoriasis compared with healthy controls (Rateb et al., 2012; Duarte et al., 2015; Zablotna et al., 2016). However, in a recent case-control study from northern India, serum RANTES levels were significantly reduced in 40 patients with chronic plaque psoriasis compared with 25 healthy controls (Joshi et al., 2019). Previous studies on the relationship between RANTES and psoriasis were observational, difficult to exclude potential confounding factors, and the sample size was small. In this study, we performed a two-sample MR using large sample size GWAS data and found that high RANTES expression was associated with increased risk of psoriasis vulgaris, indicating the positive role of the genetically predicted RANTES for psoriasis vulgaris risk.

We consistently found that psoriasis vulgaris affects the circulating levels of IL-1ra, IL-5, and MIG, which are consistent with previous findings. Abundant IL-ra expression was found in psoriatic lesions (Hammerberg et al., 1992). IL-ra expression was elevated in peripheral blood mononuclear cells of psoriasis patients compared with controls (Kim et al., 2016), while serum IL-5 levels were significantly elevated in psoriatic patients and were significantly overexpressed during the active phase of psoriasis vulgaris (Dong et al., 2021). MIG is induced by IFN-γ and can induce T cells by chemotaxis (Duarte et al., 2015). Studies showed that MIG was highly expressed in psoriatic patients and mediated T cell aggregation and activation (Goebeler et al., 1998; Duarte et al., 2015).

Compared with traditional observational studies, our MR study overcomes the bias caused by confounding and reverse causality issues and provides more reliable evidence for assessing the causal relationship between circulating cytokine concentrations and the risk of psoriasis vulgaris. However, there are some limitations in our MR study, which may influence the causal inference between cytokines and psoriasis vulgaris. Firstly, the sample size of the cytokine GWAS was limited, limiting the statistical power of causal assessment in MR. This may explain IL-17, IL-23, and TNF-α whose biologics are effective for treating psoriasis but did not yield statistically significant results in our MR analysis. Secondly, we used the significance *p*-value threshold at 1 × 10^–6^ instead of 5 × 10^–8^. The lenient *P*-value threshold increased the number of available IVs for MR but might cause weak IV bias. Thirdly, our MR study could only demonstrate the lifetime effects of higher cytokines on psoriasis vulgaris, and the effect of short-term regulation of cytokines, such as biological agents targeting cytokines, on psoriasis vulgaris is difficult to assess. Therefore, in the future studies, we need to further explore the biological functions of cytokines to better understand the relationship between cytokines and psoriasis vulgaris.

## Conclusion

We identified a causal effect of circulating RANTES levels on the risk of psoriasis vulgaris. This finding might help explore the mechanisms of RANTES in the pathogenesis and development of psoriasis and guide the evaluating of RANTES as a potential intervention target for psoriasis vulgaris.

## Data Availability

The datasets presented in this study can be found in online repositories. The names of the repository/repositories and accession number(s) can be found in the article/Supplementary Material.
