# Supplementary material for: Causal Effects of Circulating Cytokines on the Risk of Psoriasis Vulgaris: A Mendelian Randomization Study
Source: Front Genet. 2022 Jun 13;13:941961. doi: 10.3389/fgene.2022.941961 (PMC9234291; doi:10.3389/fgene.2022.941961)
Supplement: Supplementary file 1 [file Table1.DOC]

***Supplementary Material***

**Supplementary Tables**

**Table S1 Characteristics of IVs associated with each circulating cytokine level**

| **Cytokines** | **SNP ID** | **Position** | **EffectAllele/Other Allele** | **Beta** | **S.E.** | ***P***-value |
| --- | --- | --- | --- | --- | --- | --- |
|
| **RANTES** | rs112072646 | chr2:53444393 | G/A | -0.4286 | 0.0862 | 6.48E-07 |
|  | rs147509526 | chr19:15776330 | C/T | 0.358 | 0.0717 | 6.93E-07 |
|  | rs188335145 | chr2:232564462 | C/T | 0.7396 | 0.1457 | 5.76E-07 |
|  | rs2251660 | chr17:34252537 | C/A | -0.1829 | 0.0359 | 3.83E-07 |
|  | rs7000423 | chr8:111053649 | C/T | 0.1318 | 0.0253 | 1.82E-07 |
|  | rs74472919 | chr13:82200650 | C/T | -0.3313 | 0.0605 | 3.97E-08 |
| SDF-1α | rs10013755 | chr4:22197308 | T/A | -0.5302 | 0.1 | 1.50E-07 |
|  | rs141683432 | chr19:38778569 | C/T | -0.5271 | 0.1317 | 9.84E-07 |
|  | rs80202043 | chr16:88909700 | G/C | -0.1732 | 0.0387 | 3.53E-07 |
| MIP-1β | rs1005107 | chr17:34340592 | C/T | 0.1436 | 0.0261 | 3.72E-08 |
|  | rs11080371 | chr17:34375136 | C/T | -0.1035 | 0.0164 | 3.20E-10 |
|  | rs111942332 | chr17:34818508 | G/T | 0.4727 | 0.0573 | 1.70E-16 |
|  | rs112078619 | chr17:33836531 | G/A | -0.2675 | 0.0486 | 5.46E-08 |
|  | rs112257251 | chr3:45852405 | G/A | 0.1211 | 0.0196 | 7.66E-10 |
|  | rs113010081 | chr3:46457412 | C/T | 0.5954 | 0.0236 | 3.85E-140 |
|  | rs113699401 | chr17:33668796 | G/A | -0.1474 | 0.0212 | 3.35E-12 |
|  | rs113877493 | chr17:34812273 | C/T | 0.6124 | 0.0218 | 1.62E-173 |
|  | rs114164513 | chr3:45272343 | C/T | 0.3269 | 0.0351 | 2.09E-20 |
|  | rs116237296 | chr1:87045516 | G/A | -0.5437 | 0.1115 | 7.23E-07 |
|  | rs11651172 | chr17:34270288 | G/A | 0.1366 | 0.0245 | 2.11E-08 |
|  | rs117084209 | chr17:33541233 | G/C | -0.2498 | 0.0429 | 6.41E-09 |
|  | rs11710866 | chr3:48144829 | G/A | 0.1101 | 0.0177 | 5.05E-10 |
|  | rs11716293 | chr3:45304537 | G/C | -0.1013 | 0.0189 | 8.27E-08 |
|  | rs11718494 | chr3:46628788 | C/T | 0.14 | 0.0157 | 6.06E-19 |
|  | rs11720094 | chr3:46559911 | G/C | -0.1576 | 0.0157 | 1.09E-23 |
|  | rs117394484 | chr17:34363701 | C/T | 0.421 | 0.0783 | 7.69E-08 |
|  | rs117503347 | chr17:34363897 | C/T | -0.3158 | 0.0622 | 3.88E-07 |
|  | rs117518233 | chr17:34367358 | T/A | -0.4765 | 0.0633 | 7.97E-14 |
|  | rs117620244 | chr17:33648381 | C/T | 0.3528 | 0.0495 | 1.87E-12 |
|  | rs117990934 | chr17:35059123 | G/C | -0.261 | 0.0429 | 8.74E-10 |
|  | rs12450862 | chr17:35221374 | G/A | -0.2186 | 0.0349 | 7.42E-10 |
|  | rs12487651 | chr3:46714708 | G/C | -0.1397 | 0.0193 | 3.81E-13 |
|  | rs1252860 | chr17:35125060 | G/A | -0.1232 | 0.0176 | 2.60E-12 |
|  | rs12601380 | chr17:34904985 | C/A | 0.2159 | 0.0163 | 4.21E-40 |
|  | rs12601721 | chr17:32997313 | G/C | 0.1213 | 0.0178 | 9.54E-12 |
|  | rs13067782 | chr3:46593703 | C/T | -0.1549 | 0.0159 | 2.48E-22 |
|  | rs137933720 | chr3:43950371 | C/T | -0.2566 | 0.0469 | 4.42E-08 |
|  | rs138066517 | chr17:35157422 | C/T | -0.1951 | 0.0332 | 4.16E-09 |
|  | rs138273986 | chr3:45339947 | C/T | -0.4992 | 0.0831 | 1.63E-09 |
|  | rs138291909 | chr3:44426073 | C/T | 0.333 | 0.0686 | 8.93E-07 |
|  | rs138662258 | chr3:46949520 | G/A | -0.5178 | 0.0452 | 2.75E-30 |
|  | rs139288509 | chr17:33990715 | T/A | -0.3786 | 0.0777 | 6.12E-07 |
|  | rs140316381 | chr17:35327575 | G/T | 0.2605 | 0.0443 | 3.70E-09 |
|  | rs140599222 | chr17:35087781 | C/T | 0.623 | 0.1088 | 1.14E-08 |
|  | rs140950972 | chr17:34034912 | G/C | -0.2392 | 0.0485 | 9.15E-07 |
|  | rs143653036 | chr17:35140079 | G/A | -0.6966 | 0.0656 | 1.02E-25 |
|  | rs143733871 | chr17:34366898 | C/T | 0.7118 | 0.0652 | 1.68E-27 |
|  | rs144252109 | chr17:34226679 | G/A | -0.4275 | 0.061 | 4.82E-12 |
|  | rs144873592 | chr17:33940028 | G/T | -0.3186 | 0.0515 | 7.69E-10 |
|  | rs145583519 | chr17:33308246 | G/A | 0.4044 | 0.0607 | 3.39E-11 |
|  | rs146394760 | chr17:35247215 | C/T | 0.1517 | 0.0307 | 5.71E-07 |
|  | rs146565944 | chr17:33615173 | C/T | -0.2858 | 0.0562 | 4.81E-07 |
|  | rs146948889 | chr3:48477252 | G/A | -0.2944 | 0.0306 | 5.28E-22 |
|  | rs148048971 | chr17:34420067 | C/A | -0.5832 | 0.1057 | 3.56E-07 |
|  | rs148561432 | chr17:33831939 | G/A | 0.2703 | 0.0409 | 6.78E-11 |
|  | rs148883658 | chr17:34992469 | C/A | 0.5294 | 0.046 | 1.62E-30 |
|  | rs149088876 | chr17:33303609 | G/A | 0.1446 | 0.0273 | 1.23E-07 |
|  | rs150985521 | chr3:46269569 | C/T | -0.6347 | 0.0423 | 8.91E-51 |
|  | rs151009319 | chr17:33835587 | T/A | -0.5197 | 0.0661 | 8.36E-15 |
|  | rs1564708 | chr17:34825482 | C/T | 0.1744 | 0.0188 | 2.87E-20 |
|  | rs17661219 | chr17:32753821 | G/C | -0.092 | 0.0173 | 1.05E-07 |
|  | rs17693183 | chr17:34964290 | G/A | 0.5795 | 0.0795 | 8.93E-13 |
|  | rs17765088 | chr3:45943595 | G/C | 0.4577 | 0.0236 | 6.01E-84 |
|  | rs180894213 | chr17:34945008 | G/A | 0.6743 | 0.0923 | 3.96E-13 |
|  | rs182503250 | chr17:34958959 | C/T | 0.5037 | 0.0615 | 1.57E-15 |
|  | rs183139656 | chr3:50399645 | C/T | -0.3095 | 0.0408 | 3.41E-14 |
|  | rs185128671 | chr17:35722347 | G/A | 0.6071 | 0.0973 | 1.75E-09 |
|  | rs185307114 | chr17:33811332 | G/A | -0.4983 | 0.0672 | 3.13E-13 |
|  | rs185849923 | chr17:34940083 | G/A | 0.5822 | 0.0683 | 1.39E-17 |
|  | rs185994315 | chr3:46849563 | G/A | -0.4615 | 0.0769 | 2.66E-09 |
|  | rs1867288 | chr17:35021348 | G/C | -0.2079 | 0.0215 | 5.94E-22 |
|  | rs187974748 | chr17:34966572 | C/T | -0.7584 | 0.0552 | 2.07E-42 |
|  | rs187989634 | chr16:89681119 | C/T | -0.2383 | 0.0485 | 9.15E-07 |
|  | rs189547146 | chr17:33897451 | G/A | 0.5665 | 0.0837 | 6.45E-12 |
|  | rs189636230 | chr17:34439441 | G/T | -0.4442 | 0.0624 | 5.84E-12 |
|  | rs190073028 | chr17:33774465 | G/A | -0.3052 | 0.0541 | 8.83E-09 |
|  | rs190404148 | chr17:33024202 | G/A | -0.4242 | 0.0643 | 1.01E-10 |
|  | rs190685138 | chr3:46063688 | G/A | -0.5599 | 0.0816 | 6.51E-12 |
|  | rs190962112 | chr17:34833276 | C/T | -0.6017 | 0.0553 | 4.11E-27 |
|  | rs191103647 | chr3:43323804 | T/A | -0.2113 | 0.0419 | 4.70E-07 |
|  | rs191580517 | chr17:34982125 | G/T | 0.4533 | 0.0836 | 5.38E-08 |
|  | rs191589181 | chr17:34856057 | G/A | 0.5219 | 0.0555 | 3.37E-21 |
|  | rs191683532 | chr17:35777239 | C/T | -0.6123 | 0.1176 | 5.41E-07 |
|  | rs191801307 | chr17:35194144 | G/C | -0.4724 | 0.0778 | 1.41E-09 |
|  | rs191849120 | chr17:34936295 | G/T | 0.6046 | 0.0691 | 1.79E-18 |
|  | rs192235643 | chr17:34263381 | G/A | 0.5503 | 0.0699 | 7.34E-15 |
|  | rs1979671 | chr3:46274215 | C/T | -0.1216 | 0.017 | 7.86E-13 |
|  | rs1994089 | chr17:33516594 | C/T | 0.1116 | 0.0162 | 5.50E-12 |
|  | rs2003485 | chr3:50176589 | C/T | -0.0873 | 0.017 | 2.72E-07 |
|  | rs2049300 | chr3:47642726 | G/T | -0.1245 | 0.0178 | 3.11E-12 |
|  | rs211984 | chr17:35002306 | G/A | -0.0961 | 0.0174 | 3.83E-08 |
|  | rs2252547 | chr3:45821624 | C/T | -0.0913 | 0.0174 | 1.71E-07 |
|  | rs225285 | chr17:33931084 | G/C | -0.1297 | 0.0174 | 7.36E-14 |
|  | rs2276857 | chr3:45821687 | C/T | 0.1335 | 0.0257 | 2.08E-07 |
|  | rs2305097 | chr17:35550448 | C/A | -0.0932 | 0.0189 | 8.22E-07 |
|  | rs2385858 | chr3:46660436 | C/T | 0.1537 | 0.0159 | 5.52E-22 |
|  | rs2411190 | chr17:34992830 | G/A | -0.1822 | 0.0207 | 2.78E-18 |
|  | rs2673050 | chr3:45739807 | G/T | -0.1314 | 0.0161 | 3.14E-16 |
|  | rs295880 | chr17:35138390 | C/A | 0.0893 | 0.0164 | 5.65E-08 |
|  | rs323877 | chr3:51999463 | G/C | 0.1034 | 0.0172 | 2.05E-09 |
|  | rs35933743 | chr17:32970484 | G/T | 0.126 | 0.0239 | 1.50E-07 |
|  | rs4539969 | chr3:46932057 | G/A | -0.0953 | 0.0164 | 5.96E-09 |
|  | rs4683143 | chr3:45855172 | C/T | 0.1639 | 0.0159 | 9.28E-25 |
|  | rs4683173 | chr3:46099012 | G/A | 0.0961 | 0.0171 | 2.16E-08 |
|  | rs4795162 | chr17:35236530 | G/A | -0.1261 | 0.0158 | 1.14E-15 |
|  | rs4795931 | chr17:32841221 | G/A | 0.0857 | 0.0174 | 9.64E-07 |
|  | rs4796072 | chr17:33652270 | G/T | 0.1149 | 0.0177 | 8.12E-11 |
|  | rs4796235 | chr17:35014357 | G/A | -0.0919 | 0.0179 | 2.81E-07 |
|  | rs60516659 | chr17:34403297 | G/A | -0.2691 | 0.0248 | 3.64E-27 |
|  | rs62069151 | chr17:34986068 | G/A | -0.2901 | 0.029 | 1.96E-23 |
|  | rs62078065 | chr17:34287635 | C/T | -0.1963 | 0.0236 | 1.19E-16 |
|  | rs62079535 | chr17:33665329 | G/A | -0.238 | 0.039 | 1.05E-09 |
|  | rs62079661 | chr17:34351133 | G/T | 0.4904 | 0.0655 | 9.16E-14 |
|  | rs62081560 | chr17:34442541 | G/T | -0.1019 | 0.0196 | 3.45E-07 |
|  | rs62243190 | chr3:45524938 | C/T | -0.4572 | 0.0409 | 4.17E-29 |
|  | rs62263577 | chr3:47884003 | T/A | -0.1675 | 0.0303 | 3.36E-08 |
|  | rs6441984 | chr3:46470086 | T/A | 0.167 | 0.0162 | 5.29E-25 |
|  | rs6442126 | chr3:48525955 | T/A | 0.0845 | 0.0165 | 2.97E-07 |
|  | rs6505501 | chr17:34347238 | C/T | 0.1556 | 0.0191 | 3.71E-16 |
|  | rs6607344 | chr17:35075073 | G/A | -0.1063 | 0.0164 | 9.72E-11 |
|  | rs6766515 | chr3:46095235 | G/T | -0.0842 | 0.0163 | 2.58E-07 |
|  | rs6774882 | chr3:46491948 | T/A | 0.1301 | 0.0189 | 5.66E-12 |
|  | rs7213769 | chr17:36115166 | G/C | -0.1008 | 0.017 | 3.14E-09 |
|  | rs7221878 | chr17:36191133 | C/T | 0.3045 | 0.0463 | 7.37E-11 |
|  | rs72791296 | chr5:120950050 | C/T | -0.2369 | 0.0466 | 3.78E-07 |
|  | rs72825991 | chr17:33790987 | G/A | 0.1887 | 0.0312 | 1.07E-09 |
|  | rs72826154 | chr17:35434784 | C/T | -0.1548 | 0.0304 | 3.53E-07 |
|  | rs72828042 | chr17:33904832 | G/A | 0.3517 | 0.0611 | 3.22E-09 |
|  | rs72828084 | chr17:33991650 | C/T | -0.1204 | 0.0225 | 9.25E-08 |
|  | rs72829264 | chr17:32832526 | G/A | 0.1601 | 0.0278 | 9.38E-09 |
|  | rs72831789 | chr17:34396802 | T/A | 0.179 | 0.0312 | 6.69E-09 |
|  | rs73072664 | chr3:44526642 | C/A | -0.2603 | 0.042 | 5.46E-10 |
|  | rs74282562 | chr3:44140572 | C/T | -0.1418 | 0.0251 | 1.56E-08 |
|  | rs75203543 | chr3:45145171 | C/T | -0.302 | 0.0401 | 4.64E-14 |
|  | rs75394422 | chr3:49333079 | C/T | 0.3582 | 0.0408 | 1.83E-18 |
|  | rs75423501 | chr3:47242923 | G/A | 0.2094 | 0.0244 | 1.09E-17 |
|  | rs75485436 | chr3:47935009 | G/A | -0.3426 | 0.0283 | 1.07E-33 |
|  | rs762789 | chr3:46402627 | G/A | 0.1798 | 0.0165 | 1.25E-27 |
|  | rs7628174 | chr3:46750202 | C/A | -0.1236 | 0.0221 | 2.125E-08 |
|  | rs76776296 | chr7:115128487 | G/A | -0.2997 | 0.0598 | 5.547E-07 |
|  | rs76800447 | chr3:47352999 | G/A | 0.119 | 0.0198 | 1.978E-09 |
|  | rs76842834 | chr17:34883848 | C/T | 0.4206 | 0.0472 | 7.333E-19 |
|  | rs77861329 | chr3:52115752 | G/A | -0.1356 | 0.0225 | 1.596E-09 |
|  | rs78266901 | chr3:51431525 | G/A | -0.2079 | 0.0336 | 5.169E-10 |
|  | rs79068918 | chr3:45344715 | G/C | -0.2752 | 0.0271 | 3.202E-24 |
|  | rs79088462 | chr17:32851173 | C/T | 0.3323 | 0.0586 | 1.592E-08 |
|  | rs79205588 | chr17:35223674 | G/A | -0.155 | 0.0313 | 7.622E-07 |
|  | rs79544064 | chr17:34092703 | C/A | -0.2442 | 0.0398 | 6.208E-10 |
|  | rs8078470 | chr17:33774159 | G/A | 0.0966 | 0.0158 | 1.054E-09 |
|  | rs9330240 | chr17:34974689 | C/T | 0.4745 | 0.046 | 5.84E-25 |
|  | rs9838883 | chr3:45220135 | C/T | -0.0865 | 0.0166 | 1.637E-07 |
|  | rs9896155 | chr17:33794697 | G/A | -0.0826 | 0.0161 | 2.895E-07 |
|  | rs9910727 | chr17:35260546 | C/T | -0.6257 | 0.0892 | 4.646E-12 |
| **IL-17** | rs11640734 | chr16:89795797 | G/C | 0.1187 | 0.0241 | 0.000000535 |
|  | rs1530455 | chr3:122854899 | C/T | -0.108 | 0.0173 | 4.874E-10 |
|  | rs17106604 | chr14:78379156 | C/T | -0.1129 | 0.0225 | 6.374E-07 |
|  | rs17282552 | chr2:207973815 | C/T | 0.2001 | 0.0405 | 8.213E-07 |
| **MCP-1** | rs10744620 | chr12:3739094 | C/T | -0.0788 | 0.0161 | 9.91E-07 |
|  | rs10888395 | chr1:150762171 | C/T | 0.0814 | 0.0163 | 5.98E-07 |
|  | rs112313229 | chr3:46364860 | G/A | 0.1646 | 0.0313 | 1.43E-07 |
|  | rs116425179 | chr3:45598703 | G/A | 0.1476 | 0.0255 | 5.68E-09 |
|  | rs11720094 | chr3:46559911 | G/C | 0.1058 | 0.0157 | 1.54E-11 |
|  | rs12075 | chr1:159175354 | G/A | -0.2185 | 0.0155 | 1.44E-44 |
|  | rs12134665 | chr1:159388286 | G/T | -0.1046 | 0.0176 | 2.17E-09 |
|  | rs13092887 | chr3:45909644 | C/A | -0.1447 | 0.0274 | 1.22E-07 |
|  | rs146522229 | chr19:47798480 | C/T | 0.5976 | 0.1177 | 3.56E-07 |
|  | rs2036297 | chr3:46172903 | G/A | -0.119 | 0.016 | 1.09E-13 |
|  | rs2228467 | chr3:42906116 | C/T | 0.2637 | 0.0291 | 9.19E-20 |
|  | rs2281300 | chr1:159156285 | C/T | 0.0889 | 0.017 | 1.71E-07 |
|  | rs2288370 | chr3:42977436 | C/T | 0.1031 | 0.0163 | 2.25E-10 |
|  | rs2494250 | chr1:159278251 | G/C | -0.104 | 0.0186 | 2.10E-08 |
|  | rs2856757 | chr3:46410494 | C/A | -0.09 | 0.0171 | 1.27E-07 |
|  | rs34190208 | chr3:46736217 | C/T | -0.1076 | 0.022 | 9.91E-07 |
|  | rs35333710 | chr1:159172854 | G/A | -0.1476 | 0.0268 | 3.71E-08 |
|  | rs35359910 | chr3:46462904 | C/A | -0.1194 | 0.0203 | 3.34E-09 |
|  | rs56212190 | chr1:42168539 | C/T | -0.181 | 0.0373 | 9.85E-07 |
|  | rs75719572 | chr3:46183180 | G/C | -0.1231 | 0.0232 | 1.19E-07 |
|  | rs7632755 | chr3:46332382 | G/A | -0.2938 | 0.0316 | 1.18E-20 |
| **SCGF-β** | rs116924815 | chr19:51230733 | C/T | -0.6079 | 0.0738 | 1.74E-16 |
|  | rs139413256 | chr7:145879644 | G/A | 0.5377 | 0.1084 | 7.04E-07 |
|  | rs144724875 | chr19:51195936 | C/T | -0.5459 | 0.084 | 9.19E-11 |
|  | rs146024394 | chr12:99727134 | C/T | 0.4031 | 0.0807 | 5.46E-07 |
|  | rs17876031 | chr5:176831119 | G/A | 0.1514 | 0.0255 | 2.25E-09 |
|  | rs187503377 | chr12:104261835 | C/T | -0.9653 | 0.0973 | 1.34E-23 |
|  | rs190613886 | chr12:104052226 | G/A | -0.6063 | 0.0774 | 3.05E-15 |
|  | rs191631370 | chr12:104795367 | T/A | -0.8258 | 0.0919 | 2.01E-19 |
|  | rs191897096 | chr12:103540850 | G/A | -0.7692 | 0.143 | 2.20E-08 |
|  | rs4656185 | chr1:169476326 | G/A | -0.205 | 0.0256 | 1.16E-15 |
|  | rs62112533 | chr19:51233758 | C/T | 0.1667 | 0.0281 | 2.57E-09 |
| **IL-1ra** | rs116621133 | chr1:62762251 | T/A | 0.4991 | 0.0991 | 4.84E-07 |
|  | rs146151667 | chr2:215183869 | G/A | -0.5072 | 0.0978 | 3.36E-07 |
|  | rs1545747 | chr15:66457317 | G/T | 0.4582 | 0.0915 | 5.19E-07 |
|  | rs2019023 | chr19:55059249 | T/A | 0.1942 | 0.0394 | 7.83E-07 |
| **GCSF** | rs115256310 | chr5:71399691 | G/A | 0.6821 | 0.136 | 6.73E-07 |
|  | rs11903143 | chr2:29592460 | G/A | -0.087 | 0.0176 | 6.35E-07 |
|  | rs145756094 | chr9:29106805 | G/C | 0.7408 | 0.1479 | 5.53E-07 |
|  | rs2324653 | chr4:25057662 | G/C | 0.0808 | 0.0161 | 5.03E-07 |
|  | rs76287671 | chr19:44122296 | C/T | -0.0938 | 0.0189 | 6.92E-07 |
| **IL-2ra** | rs12722497 | chr10:6095928 | C/A | -0.6279 | 0.0485 | 1.57E-38 |
|  | rs12722588 | chr10:6060433 | C/T | 0.3412 | 0.0336 | 3.04E-24 |
|  | rs186795497 | chr10:6231785 | G/A | -0.7243 | 0.1396 | 2.67E-07 |
|  | rs34507893 | chr10:6124598 | G/A | -0.7344 | 0.1164 | 10 6124598 |
| **IL-18** | rs115267715 | chr5:68535015 | C/T | -0.4508 | 0.08 | 1.72E-08 |
|  | rs116383510 | chr5:2545650 | C/A | 0.5426 | 0.1056 | 3.00E-07 |
|  | rs141091241 | chr11:112111460 | C/T | 0.4122 | 0.0728 | 1.83E-08 |
|  | rs143253577 | chr5:71102576 | G/C | 0.4824 | 0.0894 | 7.44E-08 |
|  | rs150005227 | chr5:68224443 | C/T | -0.4349 | 0.0889 | 8.89E-07 |
|  | rs1534115 | chr11:112210999 | G/A | 0.149 | 0.0252 | 3.47E-09 |
|  | rs17229943 | chr5:68682536 | C/A | 0.312 | 0.0463 | 1.62E-11 |
|  | rs192929104 | chr5:68516873 | G/A | 0.9013 | 0.1499 | 3.14E-09 |
|  | rs1979967 | chr15:79659613 | C/T | -0.1402 | 0.0286 | 9.45E-07 |
|  | rs385076 | chr2:32489851 | C/T | 0.2432 | 0.0248 | 1.66E-22 |
|  | rs4482818 | chr4:65928497 | G/A | -0.1286 | 0.0244 | 1.45E-07 |
|  | rs55772679 | chr11:112235908 | C/T | 0.1393 | 0.024 | 6.86E-09 |
|  | rs658805 | chr6:70909073 | G/A | -0.1226 | 0.0244 | 4.94E-07 |
|  | rs71478720 | chr11:112009605 | C/T | 0.2669 | 0.0276 | 3.71E-22 |
|  | rs78623212 | chr7:103307627 | C/T | -0.8705 | 0.1778 | 6.71E-07 |
| **IL-2** | rs13412535 | chr2:224874874 | G/A | -0.1764 | 0.0332 | 1.18E-07 |
|  | rs1545747 | chr15:66457317 | G/T | 0.4776 | 0.0922 | 1.72E-07 |
|  | rs79992897 | chr2:224191678 | G/C | -0.9461 | 0.1833 | 2.55E-07 |
| **TRAIL** | rs112324482 | chr18:29534956 | G/A | -0.1039 | 0.0174 | 2.21E-09 |
|  | rs112452274 | chr18:28281949 | G/A | -0.8502 | 0.0632 | 5.74E-41 |
|  | rs116467561 | chr3:172529934 | C/T | 0.2099 | 0.035 | 1.81E-09 |
|  | rs11664028 | chr18:30416953 | G/T | 0.1827 | 0.0344 | 9.79E-08 |
|  | rs117153803 | chr18:28735908 | G/T | -0.1736 | 0.0295 | 4.90E-09 |
|  | rs117618570 | chr18:29008594 | G/T | 0.5294 | 0.0423 | 5.62E-36 |
|  | rs117637258 | chr18:29871632 | C/T | -0.354 | 0.0345 | 1.71E-24 |
|  | rs13278062 | chr8:23082971 | G/T | -0.0801 | 0.0157 | 3.57E-07 |
|  | rs138987090 | chr18:30366247 | G/A | 0.7497 | 0.0752 | 4.50E-23 |
|  | rs146116110 | chr18:25179662 | G/A | 0.4212 | 0.0665 | 2.30E-10 |
|  | rs150207604 | chr18:29336911 | C/A | 0.3671 | 0.0324 | 1.01E-29 |
|  | rs16962448 | chr18:29367900 | G/C | 0.1128 | 0.0182 | 5.17E-10 |
|  | rs1789041 | chr18:28555982 | G/T | -0.1254 | 0.0252 | 5.49E-07 |
|  | rs181124723 | chr3:172068242 | G/C | -0.3815 | 0.0451 | 3.75E-17 |
|  | rs183815186 | chr18:25336963 | T/A | 0.3564 | 0.0604 | 3.52E-09 |
|  | rs186728505 | chr18:33807611 | G/A | -0.3727 | 0.0813 | 9.44E-07 |
|  | rs188740444 | chr18:25126608 | G/A | -0.4904 | 0.0703 | 3.26E-12 |
|  | rs189585165 | chr18:30076725 | G/T | 0.3096 | 0.0514 | 1.65E-09 |
|  | rs193112415 | chr18:28835120 | C/T | 1.0421 | 0.0623 | 2.15E-62 |
|  | rs3136594 | chr3:172230584 | G/A | 0.1361 | 0.0171 | 1.88E-15 |
|  | rs35984124 | chr18:29357301 | G/C | -0.2464 | 0.0277 | 6.14E-19 |
|  | rs477251 | chr3:172162837 | C/T | -0.1497 | 0.0279 | 7.94E-08 |
|  | rs56011183 | chr3:171996543 | C/T | 0.2845 | 0.0398 | 1.56E-12 |
|  | rs57396456 | chr18:27945877 | C/T | 0.5626 | 0.0518 | 1.25E-27 |
|  | rs62093514 | chr18:29230977 | C/T | -1.0618 | 0.0552 | 6.86E-82 |
|  | rs62093946 | chr18:29655791 | C/T | 1.5657 | 0.0646 | 3.49E-128 |
|  | rs6764884 | chr3:172253678 | G/T | 0.1297 | 0.0227 | 9.96E-09 |
|  | rs680321 | chr18:29797958 | C/T | -0.1117 | 0.0158 | 1.57E-12 |
|  | rs7229599 | chr:27785170 | C/T | -0.1431 | 0.027 | 1.14E-07 |
|  | rs72918954 | chr18:28100226 | G/A | -0.159 | 0.0269 | 3.95E-09 |
|  | rs72965213 | chr18:32028143 | C/T | -0.3636 | 0.0566 | 9.37E-11 |
|  | rs73173340 | chr3:172390818 | G/A | 0.3146 | 0.0494 | 1.94E-10 |
|  | rs76100852 | chr18:29404330 | C/T | -0.4336 | 0.0369 | 7.72E-32 |
|  | rs78682108 | chr3:172349490 | G/A | 0.2406 | 0.0395 | 1.13E-09 |
|  | rs79287178 | chr3:172294500 | G/A | 0.4317 | 0.0421 | 9.12E-25 |
|  | rs9849338 | chr3:172126476 | C/A | 0.0797 | 0.0161 | 7.65E-07 |
|  | rs9952273 | chr18:29575063 | C/T | -0.864 | 0.0499 | 3.86E-69 |
| **MIG** | rs1796085 | chr7:70648713 | C/A | 0.2096 | 0.0403 | 2.22E-07 |
|  | rs182692989 | chr4:76736610 | C/T | 0.4717 | 0.0958 | 8.91E-07 |
|  | rs41272086 | chr6:161008646 | G/A | 0.2226 | 0.0415 | 7.43E-08 |
|  | rs62562991 | chr9:98736059 | G/A | -0.6236 | 0.126 | 8.40E-07 |
|  | rs6679677 | chr1:114303808 | C/A | -0.162 | 0.0329 | 8.86E-07 |
|  | rs816960 | chr13:108522521 | C/T | 0.1224 | 0.0244 | 5.01E-07 |
| **IL-4** | rs10512267 | chr9:102190129 | C/T | 0.0824 | 0.0161 | 2.94E-07 |
|  | rs13106889 | chr4:175960753 | T/A | 0.1161 | 0.0225 | 2.27E-07 |
|  | rs17713451 | chr7:151162472 | G/A | -0.1274 | 0.0253 | 4.97E-07 |
|  | rs73023729 | chr6:159654030 | G/A | 0.1796 | 0.0366 | 9.03E-07 |
|  | rs9941733 | chr20:374061 | G/A | -0.114 | 0.0229 | 6.88E-07 |
| **IFNg** | rs10761731 | chr10:65027610 | T/A | 0.0826 | 0.0167 | 8.09E-07 |
|  | rs113600793 | chr17:45461461 | C/A | -0.1829 | 0.0373 | 8.95E-07 |
|  | rs116609577 | chr4:169720382 | C/T | -0.2726 | 0.0544 | 5.38E-07 |
|  | rs147378920 | chr1:22701995 | G/A | 0.3863 | 0.0754 | 3.45E-07 |
|  | rs45498698 | chr1:22895820 | G/A | -0.3844 | 0.0676 | 1.13E-08 |
| **TNF-α** | rs111332265 | chr5:150393107 | G/A | 0.3766 | 0.0754 | 6.63E-07 |
|  | rs115669577 | chr4:124361448 | G/A | -0.9889 | 0.1995 | 8.28E-07 |
|  | rs188618085 | chr1:28675031 | C/T | 0.9656 | 0.195 | 7.27E-07 |
| **CTACK** | rs117500816 | chr9:33730117 | T/A | -0.2205 | 0.0451 | 9.58E-07 |
|  | rs135564 | chr22:46535934 | G/A | 0.1893 | 0.0268 | 2.43E-12 |
|  | rs145111967 | chr9:34588596 | C/T | 0.4237 | 0.0739 | 1.09E-08 |
|  | rs185804635 | chr9:32574056 | T/A | -0.6457 | 0.1324 | 6.08E-07 |
|  | rs188654671 | chr9:31504673 | C/T | -0.7017 | 0.1376 | 2.97E-07 |
|  | rs2070074 | chr9:34649442 | G/A | -0.4467 | 0.0374 | 1.79E-32 |
|  | rs2731674 | chr5:176839890 | G/T | 0.1333 | 0.0267 | 5.64E-07 |
|  | rs55764737 | chr15:61323414 | C/T | -0.5313 | 0.0972 | 4.62E-08 |
|  | rs57338032 | chr15:78798939 | G/A | -0.1583 | 0.0317 | 6.23E-07 |
|  | rs74816409 | chr9:34632067 | C/T | 0.5193 | 0.1037 | 5.80E-07 |
|  | rs76395525 | chr15:79741391 | G/A | -0.5277 | 0.1083 | 9.55E-07 |
|  | rs867811 | chr9:34663798 | C/T | -0.1908 | 0.026 | 2.37E-13 |
| **IL-5** | rs189580225 | chr14:54974461 | T/A | -0.8096 | 0.1552 | 2.26E-07 |
|  | rs73040130 | chr19:36746190 | C/T | -0.2638 | 0.0529 | 6.00E-07 |
|  | rs7767396 | chr6:43927050 | G/A | -0.1515 | 0.0246 | 7.69E-10 |
| **TNF-β** | rs76225863 | chr1:22653595 | G/A | -0.7742 | 0.123 | 1.08E-10 |
|  | rs78296352 | chr1:22821844 | G/T | -1.2215 | 0.1366 | 4.76E-21 |
|  | rs78655189 | chr1:23046887 | C/T | -1.6517 | 0.1487 | 2.93E-32 |
| **MIF** | rs12594190 | chr15:25036455 | G/A | -0.1355 | 0.0267 | 3.70E-07 |
|  | rs13142904 | chr4:54318414 | C/T | 0.223 | 0.0425 | 2.56E-07 |
|  | rs2330634 | chr22:24250795 | G/C | -0.1556 | 0.0249 | 4.53E-10 |
|  | rs78098071 | chr5:163309739 | C/T | 0.4867 | 0.0918 | 1.78E-07 |
|  | rs78434584 | chr18:22195902 | G/A | -0.5077 | 0.1018 | 5.81E-07 |
| **MCSF** | rs45498698 | chr1:22895820 | G/A | -0.617 | 0.1163 | 8.78E-08 |
|  | rs56367447 | chr8:3871527 | C/T | 0.4967 | 0.0883 | 1.72E-08 |
|  | rs62294910 | chr3:182198339 | G/A | -0.3431 | 0.0691 | 6.82E-07 |
| **IL-7** | rs144701438 | chr18:63960405 | G/A | 0.4819 | 0.0989 | 9.75E-07 |
|  | rs1539508 | chr6:43868986 | G/A | -0.1343 | 0.0257 | 2.22E-07 |
|  | rs181031888 | chr6:43927442 | T/A | -0.3883 | 0.0702 | 3.31E-08 |
|  | rs182429465 | chr6:52255687 | C/T | 0.5444 | 0.098 | 2.67E-08 |
|  | rs4320361 | chr6:43928511 | G/T | 0.3245 | 0.0249 | 6.87E-39 |
|  | rs62006410 | chr14:103007935 | C/T | 0.1557 | 0.0303 | 3.39E-07 |
| **IL-13** | rs117795020 | chr9:90084152 | G/A | 0.3522 | 0.0716 | 9.86E-07 |
|  | rs13209117 | chr6:44151765 | G/A | -0.1412 | 0.0286 | 6.98E-07 |
|  | rs143072171 | chr6:43933539 | C/T | -0.3671 | 0.0626 | 4.46E-09 |
|  | rs1539508 | chr6:43868986 | G/A | -0.1454 | 0.0252 | 1.14E-08 |
|  | rs1776721 | chr6:43890983 | G/T | 0.1676 | 0.0266 | 2.52E-10 |
|  | rs181031888 | chr6:43927442 | T/A | -0.5118 | 0.0696 | 2.74E-13 |
|  | rs192035884 | chr14:29555511 | T/A | -0.8632 | 0.1697 | 3.09E-07 |
|  | rs67798973 | chr6:43882777 | G/A | -0.1298 | 0.0248 | 1.28E-07 |
|  | rs7757246 | chr6:44003982 | C/T | -0.2171 | 0.0423 | 3.53E-07 |
|  | rs910604 | chr6:43953671 | G/A | 0.1645 | 0.0291 | 1.87E-08 |
|  | rs9472168 | chr6:43928985 | G/A | -0.4244 | 0.0248 | 1.08E-65 |
| **IP-10** | rs113831257 | chr4:76159521 | G/A | -0.3592 | 0.0644 | 2.53E-08 |
|  | rs141053179 | chr4:77589911 | G/C | 1.1031 | 0.1397 | 2.81E-15 |
|  | rs397816 | chr22:22728326 | C/T | -0.1237 | 0.0249 | 7.90E-07 |
|  | rs79848609 | chr15:87316165 | C/A | -0.2603 | 0.0537 | 8.75E-07 |
|  | rs9450351 | chr6:86624320 | C/T | 0.2768 | 0.0489 | 1.48E-08 |
| **PDGFbb** | rs112876855 | chr15:102086186 | G/A | -0.2271 | 0.0407 | 2.20E-08 |
|  | rs113685646 | chr2:224689693 | G/A | -0.3404 | 0.0557 | 1.01E-09 |
|  | rs116445074 | chr5:51534600 | G/T | -0.2931 | 0.0587 | 3.11E-07 |
|  | rs12289510 | chr11:124947051 | G/A | 0.078 | 0.0158 | 7.69E-07 |
|  | rs13024765 | chr2:225180219 | C/T | 0.1014 | 0.0158 | 1.14E-10 |
|  | rs13027940 | chr2:224895301 | C/T | 0.1458 | 0.0219 | 2.66E-11 |
|  | rs13412535 | chr2:224874874 | G/A | -0.3352 | 0.0214 | 2.46E-55 |
|  | rs1439932 | chr2:224365894 | C/T | 0.1065 | 0.0185 | 8.51E-09 |
|  | rs184996054 | chr2:225590448 | C/T | -0.2718 | 0.0513 | 1.40E-07 |
|  | rs2324229 | chr6:83918131 | C/T | -0.0894 | 0.0161 | 3.48E-08 |
|  | rs34131731 | chr2:225127338 | T/A | -0.2164 | 0.0412 | 1.38E-07 |
|  | rs4965869 | chr15:101990320 | C/T | -0.184 | 0.0181 | 5.66E-24 |
|  | rs72777070 | chr2:9798877 | G/T | 0.1069 | 0.02 | 8.98E-08 |
|  | rs78903415 | chr2:224382517 | G/C | 0.1997 | 0.0367 | 4.97E-08 |
|  | rs9941733 | chr20:374061 | G/A | -0.1161 | 0.0228 | 3.31E-07 |
| **SCF** | rs13412535 | chr2:224874874 | G/A | 0.1067 | 0.0213 | 6.04E-07 |
|  | rs1557570 | chr1:169507844 | G/T | -0.1186 | 0.017 | 2.74E-12 |
|  | rs1568119 | chr8:33243197 | C/T | 0.5906 | 0.1129 | 1.24E-07 |
|  | rs4841899 | chr9:137424412 | C/T | 0.1004 | 0.0178 | 1.78E-08 |
|  | rs635634 | chr9:136155000 | C/T | 0.1032 | 0.0191 | 6.74E-08 |
|  | rs80271436 | chr9:135897770 | G/A | 0.237 | 0.0485 | 9.95E-07 |
| **HGF** | rs10007119 | chr4:3473066 | G/A | 0.0851 | 0.0164 | 2.02E-07 |
|  | rs147729542 | chr7:81366186 | G/A | 0.3865 | 0.0681 | 1.66E-08 |
|  | rs148606324 | chr2:151218042 | C/T | -0.64 | 0.1301 | 7.92E-07 |
|  | rs3748034 | chr4:3446091 | G/T | -0.1495 | 0.0234 | 1.81E-10 |
|  | rs5745687 | chr7:81359051 | C/T | 0.3072 | 0.0406 | 2.75E-14 |
|  | rs6077285 | chr20:7880890 | G/C | 0.1393 | 0.0274 | 3.20E-07 |
| **IL-12p70** | rs10761731 | chr10:65027610 | T/A | 0.1001 | 0.0162 | 6.23E-10 |
|  | rs12199215 | chr6:44026914 | C/T | -0.1278 | 0.0192 | 5.11E-11 |
|  | rs13190738 | chr6:43845981 | C/T | -0.1012 | 0.019 | 1.48E-07 |
|  | rs13209117 | chr6:44151765 | G/A | -0.1002 | 0.0186 | 5.57E-08 |
|  | rs143072171 | chr6:43933539 | C/T | -0.3248 | 0.042 | 1.55E-14 |
|  | rs150836197 | chr6:43874928 | C/T | -0.2365 | 0.0468 | 7.74E-07 |
|  | rs1539508 | chr6:43868986 | G/A | -0.1273 | 0.0169 | 1.07E-13 |
|  | rs1776721 | chr6:43890983 | G/T | 0.1544 | 0.0172 | 1.57E-19 |
|  | rs181031888 | chr6:43927442 | T/A | -0.2849 | 0.0457 | 4.06E-10 |
|  | rs181352237 | chr6:43909740 | C/A | -0.4268 | 0.0778 | 2.24E-08 |
|  | rs2375980 | chr9:2692622 | G/C | -0.0937 | 0.0159 | 4.55E-09 |
|  | rs3025007 | chr6:43747371 | C/T | -0.0876 | 0.0171 | 3.57E-07 |
|  | rs4349809 | chr6:43924830 | G/T | -0.3777 | 0.0159 | 2.56E-124 |
|  | rs4741748 | chr9:2626851 | G/A | 0.081 | 0.0163 | 6.65E-07 |
|  | rs67798973 | chr6:43882777 | G/A | -0.1088 | 0.0161 | 8.07E-12 |
|  | rs6920532 | chr6:43793430 | C/T | -0.1596 | 0.0251 | 1.53E-10 |
|  | rs72831623 | chr17:45722293 | G/A | -0.1913 | 0.037 | 2.42E-07 |
|  | rs74556053 | chr6:43934340 | G/A | 0.2145 | 0.0351 | 6.77E-09 |
|  | rs7763358 | chr6:44139869 | C/T | -0.1562 | 0.0275 | 1.02E-08 |
|  | rs77721599 | chr6:43927526 | C/T | -0.26 | 0.0365 | 2.31E-12 |
|  | rs79873186 | chr6:43940952 | G/A | 0.2437 | 0.0376 | 8.48E-11 |
|  | rs910604 | chr6:43953671 | G/A | 0.1328 | 0.019 | 2.91E-12 |
|  | rs9381249 | chr6:43734798 | C/T | 0.1818 | 0.0368 | 5.72E-07 |
|  | rs9472183 | chr6:43940202 | G/A | 0.1019 | 0.0157 | 8.61E-11 |
| **VEGF** | rs10761731 | chr10:65027610 | T/A | 0.1187 | 0.0174 | 1.01E-11 |
|  | rs10967186 | chr9:2617099 | C/T | -0.0898 | 0.017 | 1.23E-07 |
|  | rs114181608 | chr6:43917490 | C/T | -0.2983 | 0.0469 | 1.32E-09 |
|  | rs12199215 | chr6:44026914 | C/T | -0.1517 | 0.0206 | 3.23E-13 |
|  | rs13209117 | chr6:44151765 | G/A | -0.1302 | 0.0201 | 5.28E-11 |
|  | rs143072171 | chr6:43933539 | C/T | -0.4029 | 0.044 | 2.63E-20 |
|  | rs143479231 | chr3:193110794 | G/A | 0.2598 | 0.0491 | 1.90E-07 |
|  | rs144820908 | chr6:44513485 | C/A | 0.2053 | 0.0441 | 9.94E-07 |
|  | rs150836197 | chr6:43874928 | C/T | -0.3647 | 0.0503 | 9.52E-13 |
|  | rs1539508 | chr6:43868986 | G/A | -0.1311 | 0.0182 | 1.45E-12 |
|  | rs1776721 | chr6:43890983 | G/T | 0.1883 | 0.0187 | 5.46E-24 |
|  | rs181031888 | chr6:43927442 | T/A | -0.3788 | 0.05 | 4.71E-14 |
|  | rs181352237 | chr6:43909740 | C/A | -0.5351 | 0.0836 | 7.06E-11 |
|  | rs184944915 | chr6:44023187 | G/A | 0.3411 | 0.0688 | 6.58E-07 |
|  | rs1950506 | chr6:44024262 | G/A | -0.1034 | 0.0179 | 1.02E-08 |
|  | rs3025007 | chr6:43747371 | C/T | -0.1022 | 0.0184 | 3.39E-08 |
|  | rs3025020 | chr6:43749110 | C/T | 0.1254 | 0.0254 | 8.01E-07 |
|  | rs34881325 | chr9:2622134 | C/T | 0.1082 | 0.0189 | 1.04E-08 |
|  | rs41282660 | chr6:44197006 | G/A | 0.1613 | 0.0263 | 1.33E-09 |
|  | rs4741748 | chr9:2626851 | G/A | 0.0904 | 0.0176 | 2.65E-07 |
|  | rs67798973 | chr6:43882777 | G/A | -0.1389 | 0.0175 | 1.29E-15 |
|  | rs6920532 | chr6:43793430 | C/T | -0.1803 | 0.0267 | 8.68E-12 |
|  | rs6921438 | chr6:43925607 | G/A | 0.49 | 0.0175 | 2.09E-171 |
|  | rs7030781 | chr9:2686273 | T/A | -0.1368 | 0.0173 | 2.57E-15 |
|  | rs74556053 | chr6:43934340 | G/A | 0.261 | 0.0388 | 1.58E-10 |
|  | rs7757246 | chr6:44003982 | C/T | -0.1754 | 0.0309 | 2.70E-08 |
|  | rs7763358 | chr6:44139869 | C/T | -0.2055 | 0.0294 | 1.58E-12 |
|  | rs77721599 | chr6:43927526 | C/T | -0.3016 | 0.0388 | 1.33E-14 |
|  | rs79873186 | chr6:43940952 | G/A | 0.3051 | 0.0408 | 7.64E-14 |
|  | rs8045833 | chr16:88575439 | G/A | -0.108 | 0.0211 | 2.83E-07 |
|  | rs910604 | chr6:43953671 | G/A | 0.1726 | 0.0206 | 4.87E-17 |
|  | rs9367182 | chr6:43979345 | C/T | 0.1746 | 0.0283 | 4.44E-10 |
|  | rs9381249 | chr6:43734798 | C/T | 0.2482 | 0.0397 | 3.09E-10 |
|  | rs9472183 | chr6:43940202 | G/A | 0.1282 | 0.017 | 5.19E-14 |
| **FGF-basic** | rs13412535 | chr2:224874874 | G/A | 0.1112 | 0.0225 | 7.35E-07 |
|  | rs145577605 | chr6:27610011 | G/A | -0.2081 | 0.0428 | 9.64E-07 |
|  | rs9907295 | chr17:34257313 | C/T | 0.1319 | 0.0269 | 7.95E-07 |
| **IL-6** | rs113207090 | chr14:92259809 | C/T | -0.3924 | 0.0777 | 3.69E-07 |
|  | rs13412535 | chr2:224874874 | G/A | 0.1164 | 0.0215 | 7.34E-08 |
|  | rs72831623 | chr17:45722293 | G/A | -0.1973 | 0.0372 | 1.08E-07 |
|  | rs73273528 | chr20:50431113 | C/T | -0.2672 | 0.0553 | 9.58E-07 |
| **Eotaxin** | rs11087905 | chr21:25505329 | C/A | -0.0941 | 0.0189 | 5.48E-07 |
|  | rs12075 | chr1:159175354 | G/A | -0.1671 | 0.0156 | 1.33E-26 |
|  | rs187131 | chr1:91504337 | G/C | -0.1291 | 0.0254 | 3.57E-07 |
|  | rs189587938 | chr3:46984503 | G/A | -0.3374 | 0.0668 | 1.83E-07 |
|  | rs190198015 | chr3:43131772 | C/T | -0.4281 | 0.0786 | 5.63E-08 |
|  | rs2024050 | chr7:75460393 | G/A | -0.1728 | 0.0303 | 1.10E-08 |
|  | rs2211994 | chr21:18047593 | C/T | -0.0885 | 0.0177 | 6.08E-07 |
|  | rs2228467 | chr3:42906116 | C/T | 0.4163 | 0.0292 | 2.27E-46 |
|  | rs3091309 | chr3:46303184 | G/A | -0.1283 | 0.0203 | 3.63E-10 |
|  | rs342511 | chr3:42578509 | G/A | -0.0927 | 0.0157 | 3.60E-09 |
|  | rs3746082 | chr19:39888511 | G/A | 0.322 | 0.0639 | 5.46E-07 |
|  | rs3774400 | chr3:42598407 | G/T | 0.1095 | 0.0165 | 2.85E-11 |
|  | rs3823884 | chr7:75544455 | C/A | -0.0909 | 0.0182 | 5.32E-07 |
|  | rs55946409 | chr3:42993875 | G/A | 0.1517 | 0.0164 | 2.08E-20 |
|  | rs73072941 | chr3:43307535 | T/A | 0.1309 | 0.0264 | 6.92E-07 |
|  | rs73085348 | chr3:42711221 | G/A | 0.4781 | 0.0778 | 3.85E-10 |
|  | rs7433284 | chr3:42872590 | G/A | 0.161 | 0.0165 | 1.61E-22 |
|  | rs9317045 | chr13:59630038 | C/A | -0.1182 | 0.0237 | 5.82E-07 |
| **GRO-α** | rs12047264 | chr1:159535626 | G/A | 0.1592 | 0.0291 | 6.64E-08 |
|  | rs12075 | chr1:159175354 | G/A | -0.3751 | 0.0237 | 1.24E-55 |
|  | rs13435687 | chr4:74727331 | C/T | -0.2267 | 0.031 | 3.17E-13 |
|  | rs141291554 | chr1:159225507 | C/A | -0.2603 | 0.0473 | 2.42E-08 |
|  | rs185768063 | chr6:16494983 | G/A | -0.3998 | 0.076 | 1.46E-07 |
|  | rs508977 | chr4:74762383 | G/T | 0.3802 | 0.028 | 7.56E-42 |
|  | rs72855280 | chr4:74835131 | C/A | -0.4782 | 0.0652 | 1.91E-13 |
|  | rs75078633 | chr4:75032950 | C/T | -0.9114 | 0.1837 | 7.40E-07 |
|  | rs863013 | chr1:159200020 | G/T | -0.2012 | 0.0251 | 1.12E-15 |
| **IL-16** | rs117916513 | chr11:121264274 | G/A | 0.502 | 0.0986 | 3.79E-07 |
|  | rs1255143 | chr10:130052200 | C/T | -0.1306 | 0.0242 | 7.10E-08 |
|  | rs144691581 | chr15:96953325 | G/A | -0.4882 | 0.0967 | 4.20E-07 |
|  | rs1801020 | chr5:176836532 | G/A | -0.1733 | 0.0272 | 4.53E-10 |
|  | rs190037128 | chr2:154519090 | G/A | 0.5785 | 0.1185 | 3.35E-07 |
|  | rs190994917 | chr15:81614968 | G/A | 0.8754 | 0.1526 | 1.14E-08 |
|  | rs3848180 | chr15:81596590 | G/T | -0.1223 | 0.0248 | 9.42E-07 |
|  | rs4253283 | chr4:187165211 | C/T | -0.146 | 0.0262 | 1.75E-08 |
|  | rs4513633 | chr4:113570639 | C/A | 0.2239 | 0.0453 | 7.44E-07 |
|  | rs4778636 | chr15:81591639 | G/A | 0.7272 | 0.0633 | 1.11E-30 |
| **IL-10** | rs10457128 | chr6:106017976 | G/A | 0.0865 | 0.0172 | 5.24E-07 |
|  | rs10493718 | chr1:83062933 | C/A | 0.11 | 0.0222 | 7.16E-07 |
|  | rs143072171 | chr6:43933539 | C/T | -0.2122 | 0.043 | 9.27E-07 |
|  | rs1776721 | chr6:43890983 | G/T | 0.1272 | 0.0178 | 5.25E-13 |
|  | rs181031888 | chr6:43927442 | T/A | -0.2543 | 0.0473 | 6.90E-08 |
|  | rs2375980 | chr9:2692622 | G/C | -0.0824 | 0.0165 | 7.07E-07 |
|  | rs282258 | chr2:224914800 | C/T | -0.0992 | 0.0162 | 1.00E-09 |
|  | rs4349809 | chr6:43924830 | G/T | -0.2853 | 0.0165 | 5.77E-67 |
|  | rs7088799 | chr10:65016174 | G/T | 0.0852 | 0.0167 | 3.23E-07 |
|  | rs910604 | chr6:43953671 | G/A | 0.1002 | 0.0196 | 3.45E-07 |

**Table S2** Causal effects of the rest 31 cytokines on the risk of psoriasis vulgaris in our analyses

| **Cytokines** | **No. of SNPs** | **Association** | | | **Heterogeneity** | **MR-PRESSO Global test** |
| --- | --- | --- | --- | --- | --- | --- |
|  |  | ***β*** | **S.E.** | ***P*-value** | ***P*-value** | ***P*-value** |
| **MCP-1** |  |  |  |  |  |  |
| IVW | 21 | 0.111 | 0.073 | 0.125 | 0.72 |  |
| Weighted median | 21 | 0.160 | 0.100 | 0.109 |  |  |
| MR-PRESSO | 21 | 0.111 | 0.065 | 0.102 |  | 0.710 |
| **SCGF-β** |  |  |  |  |  |  |
| IVW | 11 | -0.108 | 0.073 | 0.138 | 0.74 |  |
| Weighted median | 11 | -0.109 | 0.096 | 0.256 |  |  |
| MR-PRESSO | 11 | -0.108 | 0.060 | 0.103 |  | 0.693 |
| **IL-1ra** |  |  |  |  |  |  |
| IVW | 4 | 0.248 | 0.170 | 0.144 | 0.37 |  |
| Weighted median | 4 | 0.233 | 0.205 | 0.256 |  |  |
| MR-PRESSO | 4 | 0.248 | 0.170 | 0.241 |  | 0.411 |
| **GCSF** |  |  |  |  |  |  |
| IVW | 5 | -0.165 | 0.120 | 0.171 | 0.80 |  |
| Weighted median | 5 | -0.169 | 0.154 | 0.271 |  |  |
| MR-PRESSO | 5 | -0.165 | 0.077 | 0.100 |  | 0.799 |
| **IL-2ra** |  |  |  |  |  |  |
| IVW | 4 | 0.103 | 0.076 | 0.178 | 0.55 |  |
| Weighted median | 4 | 0.104 | 0.078 | 0.181 |  |  |
| MR-PRESSO | 4 | 0.103 | 0.064 | 0.206 |  | 0.737 |
| **IL-18** |  |  |  |  |  |  |
| IVW | 15 | 0.081 | 0.060 | 0.179 | 0.88 |  |
| Weighted median | 15 | 0.007 | 0.082 | 0.936 |  |  |
| MR-PRESSO | 15 | 0.081 | 0.046 | 0.101 |  | 0.875 |
| **IL-2** |  |  |  |  |  |  |
| IVW | 3 | 0.235 | 0.177 | 0.184 | 0.68 |  |
| Weighted median | 3 | 0.228 | 0.217 | 0.293 |  |  |
| MR-PRESSO | 3 |  |  |  |  |  |
| **TRAIL** |  |  |  |  |  |  |
| IVW | 37 | 0.071 | 0.058 | 0.220 | 0.00 |  |
| Weighted median | 37 | -0.005 | 0.064 | 0.943 |  |  |
| MR-PRESSO | 37 | NA | NA | NA |  | 0.003 |
| **MIG** |  |  |  |  |  |  |
| IVW | 6 | 0.127 | 0.122 | 0.296 | 0.95 |  |
| Weighted median | 6 | 0.133 | 0.149 | 0.373 |  |  |
| MR-PRESSO | 6 | 0.127 | 0.059 | 0.082 |  | 0.957 |
| **IL-4** |  |  |  |  |  |  |
| IVW | 5 | -0.204 | 0.200 | 0.309 | 0.95 |  |
| Weighted median | 5 | -0.158 | 0.235 | 0.501 |  |  |
| MR-PRESSO | 5 | -0.204 | 0.086 | 0.078 |  | 0.954 |
| **IFNg** |  |  |  |  |  |  |
| IVW | 5 | -0.275 | 0.274 | 0.317 | 0.14 |  |
| Weighted median | 5 | -0.147 | 0.277 | 0.596 |  |  |
| MR-PRESSO | 5 | -0.275 | 0.274 | 0.374 |  | 0.164 |
| **TNF-α** |  |  |  |  |  |  |
| IVW | 3 | -0.124 | 0.135 | 0.358 | 0.61 |  |
| Weighted median | 3 | -0.119 | 0.154 | 0.438 |  |  |
| MR-PRESSO | 3 |  |  |  |  |  |
| **CTACK** |  |  |  |  |  |  |
| IVW | 13 | 0.062 | 0.074 | 0.402 | 0.38 |  |
| Weighted median | 13 | 0.096 | 0.100 | 0.339 |  |  |
| MR-PRESSO | 13 | 0.062 | 0.074 | 0.419 |  | 0.370 |
| **IL-5** |  |  |  |  |  |  |
| IVW | 3 | 0.145 | 0.176 | 0.411 | 0.92 |  |
| Weighted median | 3 | 0.148 | 0.192 | 0.439 |  |  |
| MR-PRESSO | 3 |  |  |  |  |  |
| **TNF-β** |  |  |  |  |  |  |
| IVW | 3 | -0.046 | 0.060 | 0.443 | 0.77 |  |
| Weighted median | 3 | -0.038 | 0.065 | 0.562 |  |  |
| MR-PRESSO | 3 |  |  |  |  |  |
| **MIF** |  |  |  |  |  |  |
| IVW | 5 | 0.094 | 0.142 | 0.508 | 0.75 |  |
| Weighted median | 5 | -0.014 | 0.178 | 0.938 |  |  |
| MR-PRESSO | 5 | 0.094 | 0.099 | 0.395 |  | 0.682 |
| **MCSF** |  |  |  |  |  |  |
| IVW | 3 | -0.133 | 0.215 | 0.537 | 0.05 |  |
| Weighted median | 3 | -0.060 | 0.160 | 0.710 |  |  |
| MR-PRESSO | 3 |  |  |  |  |  |
| **IL-7** |  |  |  |  |  |  |
| IVW | 6 | -0.048 | 0.089 | 0.587 | 0.36 |  |
| Weighted median | 6 | -0.076 | 0.099 | 0.443 |  |  |
| MR-PRESSO | 6 | -0.048 | 0.089 | 0.611 |  | 0.492 |
| **IL-13** |  |  |  |  |  |  |
| IVW | 11 | 0.032 | 0.062 | 0.606 | 0.48 |  |
| Weighted median | 11 | 0.046 | 0.077 | 0.548 |  |  |
| MR-PRESSO | 11 | 0.032 | 0.060 | 0.608 |  | 0.579 |
| **IP-10** |  |  |  |  |  |  |
| IVW | 5 | 0.058 | 0.138 | 0.675 | 0.24 |  |
| Weighted median | 5 | -0.049 | 0.151 | 0.746 |  |  |
| MR-PRESSO | 5 | 0.058 | 0.138 | 0.696 |  | 0.216 |
| **PDGFbb** |  |  |  |  |  |  |
| IVW | 15 | 0.034 | 0.085 | 0.694 | 0.27 |  |
| Weighted median | 15 | 0.030 | 0.103 | 0.773 |  |  |
| MR-PRESSO | 15 | 0.034 | 0.085 | 0.700 |  | 0.358 |
| **SCF** |  |  |  |  |  |  |
| IVW | 6 | -0.070 | 0.215 | 0.743 | 0.05 |  |
| Weighted median | 6 | -0.171 | 0.200 | 0.394 |  |  |
| MR-PRESSO | 6 | -0.070 | 0.215 | 0.756 |  | 0.060 |
| **HGF** |  |  |  |  |  |  |
| IVW | 6 | -0.039 | 0.138 | 0.775 | 0.56 |  |
| Weighted median | 6 | 0.067 | 0.173 | 0.700 |  |  |
| MR-PRESSO | 6 | -0.039 | 0.122 | 0.760 |  | 0.549 |
| **IL-12p70** |  |  |  |  |  |  |
| IVW | 24 | 0.015 | 0.060 | 0.806 | 0.79 |  |
| Weighted median | 24 | 0.053 | 0.084 | 0.528 |  |  |
| MR-PRESSO | 24 | 0.015 | 0.052 | 0.780 |  | 0.827 |
| **VEGF** |  |  |  |  |  |  |
| IVW | 34 | 0.010 | 0.044 | 0.818 | 0.65 |  |
| Weighted median | 34 | -0.029 | 0.066 | 0.659 |  |  |
| MR-PRESSO | 34 | 0.010 | 0.042 | 0.808 |  | 0.663 |
| **FGF-basic** |  |  |  |  |  |  |
| IVW | 3 | -0.063 | 0.276 | 0.819 | 0.88 |  |
| Weighted median | 3 | -0.050 | 0.318 | 0.874 |  |  |
| MR-PRESSO | 3 |  |  |  |  |  |
| **IL-6** |  |  |  |  |  |  |
| IVW | 4 | 0.041 | 0.196 | 0.835 | 0.53 |  |
| Weighted median | 4 | -0.062 | 0.241 | 0.797 |  |  |
| MR-PRESSO | 4 | 0.041 | 0.168 | 0.824 |  | 0.498 |
| **Eotaxin** |  |  |  |  |  |  |
| IVW | 18 | 0.014 | 0.076 | 0.849 | 0.44 |  |
| Weighted median | 18 | 0.074 | 0.106 | 0.484 |  |  |
| MR-PRESSO | 18 | 0.014 | 0.076 | 0.851 |  | 0.469 |
| **GRO-α** |  |  |  |  |  |  |
| IVW | 9 | -0.013 | 0.073 | 0.863 | 0.07 |  |
| Weighted median | 9 | -0.031 | 0.076 | 0.687 |  |  |
| MR-PRESSO | 9 | -0.013 | 0.073 | 0.867 |  | 0.080 |
| **IL-16** |  |  |  |  |  |  |
| IVW | 10 | -0.002 | 0.063 | 0.970 | 0.96 |  |
| Weighted median | 10 | 0.022 | 0.072 | 0.759 |  |  |
| MR-PRESSO | 10 | -0.002 | 0.037 | 0.950 |  | 0.943 |
| **IL-10** |  |  |  |  |  |  |
| IVW | 10 | -0.002 | 0.092 | 0.982 | 0.68 |  |
| Weighted median | 10 | 0.067 | 0.108 | 0.533 |  |  |
| MR-PRESSO | 10 | -0.002 | 0.079 | 0.979 |  | 0.627 |

**Table S3** MR Egger's regression analysis for the causal effect of cytokine levels on psoriasis vulgaris

| **Cytokines** | **Egger regression** | |
| --- | --- | --- |
|  | **Intercept** | ***P*-value** |
| **RANTES** | -0.02 | 0.69 |
| **SDF-1α** | 2.20 | 0.51 |
| **MIP-1β** | 0.00 | 0.97 |
| **IL-17** | -0.03 | 0.92 |
| MCP-1 | -0.01 | 0.68 |
| SCGF-β | 0.00 | 0.99 |
| IL-1ra | -0.11 | 0.47 |
| GCSF | 0.02 | 0.56 |
| IL-2ra | 0.02 | 0.88 |
| IL-18 | -0.01 | 0.70 |
| IL-2 | -0.08 | 0.58 |
| TRAIL | 0.01 | 0.53 |
| MIG | -0.03 | 0.59 |
| IL-4 | 0.07 | 0.57 |
| IFNg | 0.05 | 0.52 |
| TNF-α | -0.15 | 0.53 |
| CTACK | 0.01 | 0.90 |
| IL-5 | 0.02 | 0.91 |
| TNF-β | 0.15 | 0.67 |
| MIF | 0.03 | 0.73 |
| MCSF | 0.13 | 0.86 |
| IL-7 | 0.02 | 0.71 |
| IL-13 | 0.00 | 0.99 |
| IP-10 | -0.05 | 0.50 |
| PDGFbb | 0.00 | 0.89 |
| SCF | 0.08 | 0.29 |
| HGF | -0.04 | 0.44 |
| IL-12p70 | 0.00 | 0.81 |
| VEGF | 0.02 | 0.22 |
| FGF-basic | -0.09 | 0.78 |
| IL-6 | -0.08 | 0.46 |
| Eotaxin | -0.04 | 0.17 |
| GRO-α | -0.01 | 0.92 |
| IL-16 | -0.02 | 0.58 |
| IL-10 | -0.02 | 0.40 |

**Table S4** MR analysis for the causal effect of psoriasis vulgaris on cytokine levels

| **Cytokines** | **No. of SNPs** | **Association** | | | **Heterogeneity** | **MR-PRESSO Global test** |
| --- | --- | --- | --- | --- | --- | --- |
|  |  | ***β*** | **S.E.** | ***P*-value** | ***P*-value** | ***P*-value** |
| **IL-1ra** |  |  |  |  |  |  |
| IVW | 37 | 0.041 | 0.012 | **0.000** | 0.90434 |  |
| Weighted median | 37 | 0.042 | 0.018 | **0.018** |  |  |
| MR-PRESSO | 37 | 0.041 | 0.010 | **0.000** |  | 0.922 |
| **IL-5** |  |  |  |  |  |  |
| IVW | 36 | 0.043 | 0.012 | **0.000** | 0.42923 |  |
| Weighted median | 36 | 0.043 | 0.020 | **0.031** |  |  |
| MR-PRESSO | 36 | 0.043 | 0.012 | **0.001** |  | 0.460 |
| **CTACK** |  |  |  |  |  |  |
| IVW | 36 | 0.035 | 0.012 | **0.002** | 0.97656 |  |
| Weighted median | 36 | 0.026 | 0.018 | 0.135 |  |  |
| MR-PRESSO | 36 | 0.035 | 0.009 | **0.000** |  | 0.980 |
| **MIG** |  |  |  |  |  |  |
| IVW | 37 | 0.032 | 0.011 | **0.005** | 0.91592 |  |
| Weighted median | 37 | 0.039 | 0.018 | **0.028** |  |  |
| MR-PRESSO | 37 | 0.032 | 0.010 | **0.002** |  | 0.924 |
| **VEGF** |  |  |  |  |  |  |
| IVW | 37 | 0.024 | 0.008 | **0.005** | 0.33799 |  |
| Weighted median | 37 | 0.024 | 0.013 | 0.063 |  |  |
| MR-PRESSO | 37 | 0.024 | 0.008 | **0.008** |  | 0.400 |
| **IL-8** |  |  |  |  |  |  |
| IVW | 37 | 0.029 | 0.012 | **0.015** | 0.86324 |  |
| Weighted median | 37 | 0.022 | 0.019 | 0.240 |  |  |
| MR-PRESSO | 37 | 0.029 | 0.010 | **0.008** |  | 0.877 |
| **IL-9** |  |  |  |  |  |  |
| IVW | 37 | 0.028 | 0.011 | **0.016** | 0.49575 |  |
| Weighted median | 37 | 0.022 | 0.018 | 0.224 |  |  |
| MR-PRESSO | 37 | 0.028 | 0.011 | **0.021** |  | 0.538 |
| **IL-13** |  |  |  |  |  |  |
| IVW | 37 | 0.028 | 0.012 | **0.018** | 0.36710 |  |
| Weighted median | 37 | 0.029 | 0.019 | 0.119 |  |  |
| MR-PRESSO | 37 | 0.028 | 0.012 | **0.024** |  | 0.416 |
| **IL-12p70** |  |  |  |  |  |  |
| IVW | 37 | 0.016 | 0.008 | **0.037** | 0.27807 |  |
| Weighted median | 37 | 0.011 | 0.011 | 0.330 |  |  |
| MR-PRESSO | 37 | 0.016 | 0.008 | **0.044** |  | 0.307 |
| **TNF-α** |  |  |  |  |  |  |
| IVW | 37 | 0.024 | 0.012 | **0.041** | 0.55129 |  |
| Weighted median | 37 | 0.011 | 0.019 | 0.579 |  |  |
| MR-PRESSO | 37 | 0.024 | 0.012 | **0.043** |  | 0.550 |
| **IL-2** |  |  |  |  |  |  |
| IVW | 37 | 0.022 | 0.012 | 0.060 | 0.99947 |  |
| Weighted median | 37 | 0.019 | 0.018 | 0.298 |  |  |
| MR-PRESSO | 37 | 0.022 | 0.007 | **0.005** |  | 0.999 |
| **IL-17** |  |  |  |  |  |  |
| IVW | 37 | 0.017 | 0.009 | 0.056 | 0.09182 |  |
| Weighted median | 37 | 0.020 | 0.012 | 0.102 |  |  |
| MR-PRESSO | 37 | 0.017 | 0.009 | 0.064 |  | 0.108 |
| **IL-2ra** |  |  |  |  |  |  |
| IVW | 37 | 0.023 | 0.013 | 0.070 | 0.18396 |  |
| Weighted median | 37 | 0.032 | 0.018 | 0.075 |  |  |
| MR-PRESSO | 37 | 0.023 | 0.013 | 0.079 |  | 0.217 |
| **IL-1β** |  |  |  |  |  |  |
| IVW | 38 | 0.014 | 0.010 | 0.143 | 0.25591 |  |
| Weighted median | 38 | -0.002 | 0.015 | 0.899 |  |  |
| MR-PRESSO | 38 | 0.014 | 0.010 | 0.152 |  | 0.234 |
| **MCP-1** |  |  |  |  |  |  |
| IVW | 36 | 0.019 | 0.013 | 0.145 | 0.00107 |  |
| Weighted median | 36 | 0.012 | 0.014 | 0.404 |  |  |
| MR-PRESSO | 36 | 0.013 | 0.012 | 0.257 |  | 0.001 |
| **FGF-basic** |  |  |  |  |  |  |
| IVW | 37 | 0.012 | 0.009 | 0.147 | 0.17541 |  |
| Weighted median | 37 | 0.009 | 0.012 | 0.429 |  |  |
| MR-PRESSO | 37 | 0.012 | 0.009 | 0.155 |  | 0.202 |
| **IFNg** |  |  |  |  |  |  |
| IVW | 37 | 0.010 | 0.008 | 0.187 | 0.84934 |  |
| Weighted median | 37 | 0.013 | 0.012 | 0.272 |  |  |
| MR-PRESSO | 37 | 0.010 | 0.007 | 0.138 |  | 0.874 |
| **IL-10** |  |  |  |  |  |  |
| IVW | 37 | 0.009 | 0.008 | 0.223 | 0.42590 |  |
| Weighted median | 37 | 0.008 | 0.012 | 0.505 |  |  |
| MR-PRESSO | 37 | 0.009 | 0.008 | 0.231 |  | 0.475 |
| **MCSF** |  |  |  |  |  |  |
| IVW | 35 | 0.020 | 0.017 | 0.251 | 0.93483 |  |
| Weighted median | 35 | 0.027 | 0.024 | 0.260 |  |  |
| MR-PRESSO | 35 | 0.020 | 0.014 | 0.167 |  | 0.931 |
| **Eotaxin** |  |  |  |  |  |  |
| IVW | 37 | 0.009 | 0.009 | 0.301 | 0.06939 |  |
| Weighted median | 37 | -0.011 | 0.011 | 0.326 |  |  |
| MR-PRESSO | 37 | NA | NA | NA |  | 0.027 |
| **IL-18** |  |  |  |  |  |  |
| IVW | 37 | 0.013 | 0.013 | 0.318 | 0.10663 |  |
| Weighted median | 37 | 0.011 | 0.018 | 0.537 |  |  |
| MR-PRESSO | 37 | 0.013 | 0.013 | 0.325 |  | 0.132 |
| **MIP-1β** |  |  |  |  |  |  |
| IVW | 37 | 0.009 | 0.009 | 0.323 | 0.06122 |  |
| Weighted median | 37 | 0.009 | 0.011 | 0.421 |  |  |
| MR-PRESSO | 37 | 0.009 | 0.009 | 0.330 |  | 0.074 |
| **SCGF-β** |  |  |  |  |  |  |
| IVW | 37 | 0.011 | 0.011 | 0.344 | 0.98452 |  |
| Weighted median | 37 | 0.006 | 0.018 | 0.748 |  |  |
| MR-PRESSO | 37 | 0.011 | 0.009 | 0.214 |  | 0.986 |
| **MCP3** |  |  |  |  |  |  |
| IVW | 30 | 0.030 | 0.032 | 0.357 | 0.03285 |  |
| Weighted median | 30 | 0.014 | 0.037 | 0.710 |  |  |
| MR-PRESSO | 30 | NA | NA | NA |  | 0.042 |
| **HGF** |  |  |  |  |  |  |
| IVW | 37 | 0.007 | 0.007 | 0.367 | 0.44586 |  |
| Weighted median | 37 | 0.009 | 0.012 | 0.428 |  |  |
| MR-PRESSO | 37 | 0.007 | 0.007 | 0.373 |  | 0.485 |
| **GROα** |  |  |  |  |  |  |
| IVW | 37 | 0.010 | 0.012 | 0.377 | 0.88476 |  |
| Weighted median | 37 | 0.008 | 0.019 | 0.684 |  |  |
| MR-PRESSO | 37 | 0.010 | 0.010 | 0.307 |  | 0.901 |
| **GCSF** |  |  |  |  |  |  |
| IVW | 37 | 0.007 | 0.008 | 0.387 | 0.82236 |  |
| Weighted median | 37 | 0.009 | 0.011 | 0.430 |  |  |
| MR-PRESSO | 37 | 0.007 | 0.007 | 0.335 |  | 0.855 |
| **RANTES** |  |  |  |  |  |  |
| IVW | 37 | 0.011 | 0.014 | 0.408 | 0.10395 |  |
| Weighted median | 37 | 0.010 | 0.018 | 0.599 |  |  |
| MR-PRESSO | 37 | 0.011 | 0.014 | 0.414 |  | 0.121 |
| **IP-10** |  |  |  |  |  |  |
| IVW | 37 | 0.009 | 0.011 | 0.410 | 0.44670 |  |
| Weighted median | 37 | -0.008 | 0.018 | 0.655 |  |  |
| MR-PRESSO | 37 | 0.009 | 0.011 | 0.415 |  | 0.433 |
| **SDF-1α** |  |  |  |  |  |  |
| IVW | 37 | 0.007 | 0.008 | 0.413 | 0.17003 |  |
| Weighted median | 37 | 0.005 | 0.012 | 0.697 |  |  |
| MR-PRESSO | 37 | 0.007 | 0.008 | 0.419 |  | 0.207 |
| **IL-6** |  |  |  |  |  |  |
| IVW | 36 | 0.007 | 0.009 | 0.464 | 0.68031 |  |
| Weighted median | 36 | 0.007 | 0.014 | 0.590 |  |  |
| MR-PRESSO | 36 | 0.007 | 0.009 | 0.439 |  | 0.676 |
| **IL-7** |  |  |  |  |  |  |
| IVW | 37 | 0.009 | 0.012 | 0.475 | 0.62018 |  |
| Weighted median | 37 | -0.007 | 0.019 | 0.717 |  |  |
| MR-PRESSO | 37 | 0.009 | 0.011 | 0.460 |  | 0.602 |
| **β-NGF** |  |  |  |  |  |  |
| IVW | 37 | 0.008 | 0.012 | 0.478 | 0.90045 |  |
| Weighted median | 37 | 0.020 | 0.018 | 0.256 |  |  |
| MR-PRESSO | 37 | 0.008 | 0.010 | 0.406 |  | 0.905 |
| **IL-4** |  |  |  |  |  |  |
| IVW | 37 | 0.005 | 0.008 | 0.513 | 0.37141 |  |
| Weighted median | 37 | 0.001 | 0.011 | 0.931 |  |  |
| MR-PRESSO | 37 | 0.005 | 0.008 | 0.517 |  | 0.400 |
| **PDGFbb** |  |  |  |  |  |  |
| IVW | 37 | 0.004 | 0.007 | 0.586 | 0.86421 |  |
| Weighted median | 37 | 0.012 | 0.011 | 0.289 |  |  |
| MR-PRESSO | 37 | 0.004 | 0.006 | 0.533 |  | 0.849 |
| **SCF** |  |  |  |  |  |  |
| IVW | 37 | 0.004 | 0.008 | 0.607 | 0.26757 |  |
| Weighted median | 37 | -0.001 | 0.011 | 0.913 |  |  |
| MR-PRESSO | 37 | 0.004 | 0.008 | 0.610 |  | 0.306 |
| **IL-16** |  |  |  |  |  |  |
| IVW | 37 | 0.007 | 0.015 | 0.624 | 0.01212 |  |
| Weighted median | 37 | -0.001 | 0.018 | 0.974 |  |  |
| MR-PRESSO | 37 | 0.001 | 0.014 | 0.943 |  | 0.014 |
| **TRAIL** |  |  |  |  |  |  |
| IVW | 36 | 0.004 | 0.009 | 0.669 | 0.83223 |  |
| Weighted median | 36 | 0.006 | 0.013 | 0.654 |  |  |
| MR-PRESSO | 36 | 0.004 | 0.008 | 0.629 |  | 0.829 |
| **MIF** |  |  |  |  |  |  |
| IVW | 37 | 0.002 | 0.014 | 0.861 | 0.08912 |  |
| Weighted median | 37 | 0.004 | 0.019 | 0.850 |  |  |
| MR-PRESSO | 37 | 0.002 | 0.014 | 0.862 |  | 0.108 |
| **TNF-β** |  |  |  |  |  |  |
| IVW | 28 | 0.004 | 0.022 | 0.876 | 0.47189 |  |
| Weighted median | 28 | -0.023 | 0.033 | 0.482 |  |  |
| MR-PRESSO | 28 | 0.004 | 0.022 | 0.876 |  | 0.465 |

**Table S5** MR Egger's regression analysis for the causal effect of psoriasis vulgaris on cytokine levels

| **Cytokines** | **Egger regression** | |
| --- | --- | --- |
|  | **Intercept** | ***P*-value** |
| **IL-1ra** | -0.00363 | 0.65170 |
| **IL-5** | -0.00397 | 0.64359 |
| **CTACK** | 0.00455 | 0.57343 |
| **MIG** | -0.00588 | 0.46053 |
| **VEGF** | 0.00167 | 0.77857 |
| **IL-8** | 0.00034 | 0.96647 |
| **IL-9** | 0.00022 | 0.97770 |
| **IL-13** | -0.00492 | 0.56096 |
| **IL-12p70** | 0.00089 | 0.87326 |
| **TNF-α** | 0.00200 | 0.80772 |
| **IL-2** | 0.00077 | 0.92495 |
| IL-17 | -0.00132 | 0.83308 |
| IL-2ra | -0.01542 | 0.07516 |
| IL-1β | 0.00939 | 0.17198 |
| MCP-1 | -0.00130 | 0.89563 |
| FGF-basic | 0.00232 | 0.70187 |
| IFNg | -0.00049 | 0.92734 |
| IL-10 | 0.00238 | 0.66671 |
| MCSF | 0.00658 | 0.61456 |
| Eotaxin | 0.00852 | 0.16396 |
| IL-18 | -0.00279 | 0.76224 |
| MIP-1β | 0.00365 | 0.55414 |
| SCGF-β | 0.00653 | 0.41332 |
| MCP-3 | 0.01308 | 0.60009 |
| HGF | -0.00323 | 0.54053 |
| GRO-α | -0.00197 | 0.80890 |
| GCSF | 0.00247 | 0.64145 |
| RANTES | 0.00461 | 0.63069 |
| IP-10 | -0.00033 | 0.96741 |
| SDF-1α | -0.00213 | 0.72124 |
| IL-6 | 0.00058 | 0.93530 |
| IL-7 | 0.00250 | 0.76270 |
| β-NGF | -0.00444 | 0.58634 |
| IL-4 | 0.00494 | 0.36259 |
| PDGFbb | -0.00313 | 0.54576 |
| SCF | 0.00152 | 0.78528 |
| IL-16 | 0.00667 | 0.52374 |
| TRAIL | 0.01363 | 0.06210 |
| MIF | 0.00403 | 0.67506 |
| TNF-β | 0.03232 | 0.06790 |
